# Systematic Review and Meta‐Analysis of the Association Between Clinical Severity and Co‐Infection of Human Adenovirus With Other Respiratory Pathogens in Children

**DOI:** 10.1002/jmv.70370

**Published:** 2025-04-29

**Authors:** Dandan Niu, Yanxiao Gao, Yingluan Zhang, Qiuying Lv, Yiwen Jiang, Yuanxi Jia, Zhigao Chen, Honglin Wang, Yanpeng Cheng, Feng Sha, Meng Ren, Yixiong Chen, Xindong Zhang, Zhen Zhang, Jinling Tang, Tiejian Feng

**Affiliations:** ^1^ Shenzhen Institute of Advanced Technology Chinese Academy of Sciences Shenzhen China; ^2^ Department of Communicable Diseases Control and Prevention Shenzhen Center for Disease Control and Prevention Shenzhen China; ^3^ Shenzhen Research Center for Communicable Disease Control and Prevention Chinese Academy of Medical Sciences Shenzhen China; ^4^ Department of Communicable Diseases Control and Prevention Baoan District Center for Disease Control and Prevention Shenzhen China; ^5^ Faculty of Computer Science and Control Engineering Shenzhen University of Advanced Technology Shenzhen China

**Keywords:** clinical outcomes, co‐infection, human respiratory adenovirus infections

## Abstract

The correlation between the co‐infection of human respiratory adenovirus (HAdV) and clinical severity has not been firmly established yet. We carried out a systematic review and meta‐analysis. We scoured six databases for studies published up to 16 May 2024. A total of 66 cohort studies, which involved 16 251 participants, were incorporated. When compared with patients suffering from HAdV single infection, those with co‐infection of viruses (risk ratios [RRs] = 1.40, 95% confidence interval [CI]: 1.05–1.86), bacteria (RR = 1.50, 95% CI: 1.05–2.16), or fungi (RR = 2.86, 95% CI: 2.17–3.76) were more prone to experience severe clinical outcomes. Co‐infection with *Mycoplasma pneumoniae* had a tendency to elevate the risk of common pneumonia (RR = 1.81, 95% CI: 1.66–1.97), and bacterial co‐infection was likely to extend the hospital stay (mean differences = 2.23 days, 95% CI: 0.44–4.03). In summary, the co‐infection of HAdV with other viral, bacterial, fungal respiratory pathogens or *Mycoplasma pneumoniae* heightened the risk of severe clinical outcomes in pediatric patients, leading to an increased utilization of medical resources. This implied that the ecological and biological mechanisms underlying the potential interactions between HAdV and other microorganisms merited further investigation.

## 1 Introduction

Human adenovirus (HAdV) was initially isolated in 1953 by Rowe and his colleagues from adenoid tissue. In immunocompetent individuals, it typically leads to mild, self‐limiting infections. HAdV presents a diverse range of clinical manifestations, with the affected sites including the lower respiratory tract, digestive tract, and ocular tract, depending on the specific genotype. This virus mainly affects children and those in close‐contact settings. Individuals at a high risk of Human Adenovirus (HAdV) infection primarily consist of children in daycare centers, educational institutions, summer camps, and those under the age of five. In the pediatric population, approximately 5% of respiratory tract infections can be attributed to HAdV. A recent study revealed that between 6% and 20% of children hospitalized due to lower respiratory tract infections tested positive for HAdV [1]. Children aged between 6 months and 2 years enrolled in childcare were at a higher risk of experiencing severe complications from HAdV infections.

In both research and clinical settings, novel diagnostic and detection methods for respiratory pathogens have become widely accessible. The simultaneous detection of HAdV along with other respiratory pathogens has reached a frequency of up to 50% [2]. Nonetheless, the published literature revealed divergent perspectives regarding the correlation between co‐infections of other pathogens and the clinical severity in patients infected with HAdV. A meta‐analysis of nine case‐control or cohort studies found that the presence of HAdV in conjunction with other viruses was associated with an increased risk of poor prognosis in children with severe pneumonia [3], while several systematic reviews suggested that there was no association between disease severity and viral co‐infections [4]. A study in China showed that the combination of HAdV with bacterial infections did not elevate the risk of severe pneumonia [5], while a study in Vietnam revealed that the combination substantially heightened the risk of developing severe pneumonia [6].

The findings of existing reviews and individual studies were inconsistent. There is no recent meta‐analysis examining the clinical implications of HAdV co‐infection with a specific pathogen. The correlation between co‐infection of HAdV with other pathogens and clinical severity has yet to be firmly determined.

### 1.1 Objective

We aimed to perform a systematic review and meta‐analysis to assess the association between HAdV co‐infection with other pathogens and clinical outcomes (hospitalization, pneumonia severity, and deaths) in children.

## 2 Materials and Methods

The protocol of this systematic review and meta‐analysis was registered in Prospective Register of Systematic Reviews (The ID number: CRD42024547528). We reported our review using the Preferred Reporting Items for Systematic Reviews and Meta Analyses (PRISMA) guidelines, as detailed in Supporting Information S1: Table S1.

### 2.1 Literature Search

We searched six electronic databases (MEDLINE, Embase, Web of Science, China National Knowledge Infrastructure, Wanfang Data, and Chongqing VIP Information) up until 16 May 2024 without any restrictions on the starting year. Our search focused on identifying cohort studies of reporting the clinical outcomes of HAdV mono‐infection and co‐infection among outpatients or inpatients under the age of 18 years. We did not incorporate age‐specific search terms into our search strategy because studies that encompassed a broader age range (e.g., all ages) may still report on sub‐age groups that meet the criteria for inclusion in our review. Only studies published in Chinese or English were considered. Additionally, we manually examined the reference lists of the included studies to uncover further relevant research. The search strategy was outlined in Supporting Information S1: Appendix Box 1.

### 2.2 Study Selection

Eligible studies were cohort studies that reported clinical outcomes for outpatients or inpatients under 18 years old with laboratory‐confirmed mono‐ and co‐infections of HAdV. Three severe clinical outcomes were assessed: (1) Hospitalization, further categorized into general hospitalization, the length of hospital stay, and admission to intensive care unit (ICU); (2) Pneumonia severity, categorized into common pneumonia including common pneumonia and bronchiolitis obliterans (BO) and severe pneumonia including severe pneumonia, Appendix oxygen and mechanical ventilation); (3) Death. The definitions of common pneumonia and severe pneumonia were based on the 2016 clinical practice guidelines published by the Chinese Thoracic Society [7]. HAdV co‐infection was defined as the simultaneous detection of HAdV and other common or conditional pathogens.

We excluded studies that restricted the study population to patients with comorbidities or preterm infants, and that had a sample size smaller than 10. Studies that solely used serological tests to confirm infections of HAdV along with other pathogens were also excluded due to its inaccuracy.

Two researchers (D. Niu and Y. Zhang) independently screened the title, abstract and full‐text of each retrieved records, and any inconsistencies were resolved by discussion with third researcher (H. Wang).

### 2.3 Data Extraction and Study Quality Assessment

The data extracted from the included studies included general information and three clinical outcomes (hospitalization, pneumonia severity, and deaths) associated with HAdV mono‐ versus co‐infection. The general information included author, publication year, study duration, geographical location, country, study setting (community or hospital), healthcare setting (inpatient, outpatient, or emergency), age of study participant, sample size, diagnostic criteria or symptoms, specimen type, and method of pathogen detection. We calculated mean differences (MDs) or risk ratios (RRs), and their 95% confidence intervals (CIs) for assessing the relationship between clinical severity and HAdV co‐infection.

The quality assessment of cohort studies was conducted using the Newcastle‐Ottawa Scale checklist. Based on the cumulative quality score, studies were classified as high (6–9 points) or low (0–5 points) quality. Quality assessment was conducted independently by D. Niu and Y. Zhang, with any disagreements arbitrated by H. Wang.

### 2.4 Statistical Analysis

The primary outcome of the meta‐analysis was the association between three clinical outcomes and co‐infections of HAdV and any viral, bacterial, fungal or atypical pathogen. Secondary outcomes focused on infections involving a single pathogen pair. Regarding the length of hospital stay, MDs and 95% CIs were calculated from the original study outcomes. If the available raw data solely consisted of median and interquartile range (IQR), these values were converted into mean and standard deviation (SD) for analysis [8]. For other clinical outcomes, RRs and 95% CIs were calculated based on the original study results. A meta‐analysis of MDs or RRs was performed using the Mantel‐Haenszel random effects model when two or more studies were available for each coinfected pathogen and outcome. If the number of positive clinical outcomes was zero for both the HAdV mono‐ and co‐infection groups within a study, the study was described qualitatively. If the number of positive clinical outcomes was zero for either the HAdV mono‐ or co‐infection group within a study, it was substituted with 0.5.

We employed the *I* ^*2*^ statistic to evaluate statistical heterogeneity among the included studies, considering an *I* ^*2*^ value of less than 50% as indicative of low heterogeneity, 50%–75% as moderate heterogeneity, and greater than 75% as high heterogeneity. Publication bias was assessed using funnel plots, Begg's and Egger's test simultaneously. Sensitivity analysis was performed ad hoc by sequentially removing individual studies from the analysis, employing the leave‐one‐out method. A separate meta‐analysis of the original study whose clinical outcome was severe pneumonia was performed for sensitivity analysis. All statistical analyses were executed using R version 4.4.1. A *p* value less than 0.05 was deemed statistically significant except Begg's and Egger's test.

## 3 Results

**Figure 1:**
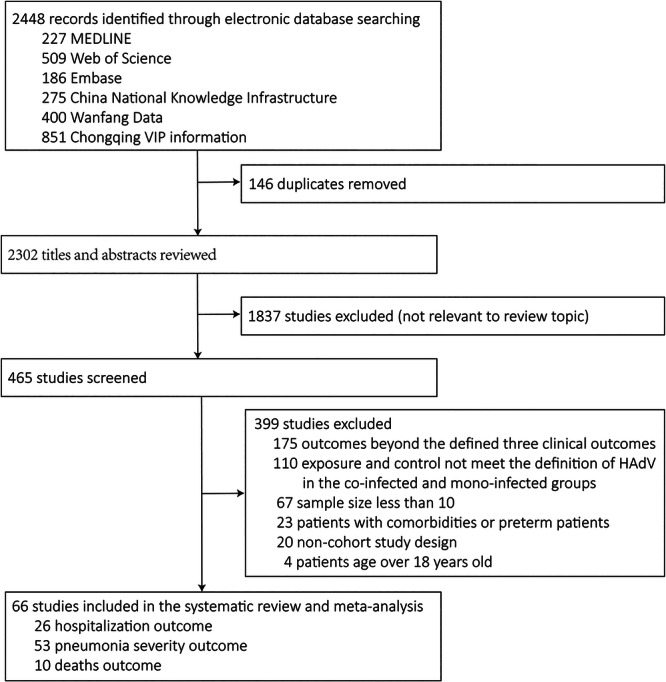
Flowchart of literature search.

Of 2448 records, we reviewed 2302 nonduplicate titles and abstracts and subsequently evaluated 465 full‐text studies, and 66 cohort studies were deemed eligible. Our systematic review and meta‐analysis encompassed 66 cohort studies with a total of 16 251 participants (Figure 1). We analyzed 26 studies that reported hospitalization outcomes, involving a total of 6247 participants; 53 studies that assessed pneumonia severity, with 14 360 participants; and 10 studies that reported deaths outcomes, including 1049 participants. The quality assessment indicated that the studies included were of high quality. Additional information and a detailed quality assessment of these studies were provided in Supporting Information S1: Tables S2–S4.

### 3.1 The Association Between Co‐Infection and Risk of Hospitalization

Four studies compared the general hospitalization rates of patients with HAdV mono‐infection against co‐infection with any virus [9, 10, 11, 12]. Fifteen studies documented the length of hospital stay for patients with HAdV mono‐infection compared to those with co‐infection (Supporting Information S1: Table S5). Seven studies documented the admission rates to ICU among patients with mono‐infection due to HAdV compared to those with co‐infection [1, 10, 13, 14, 15, 16, 17].

**Figure 2:**
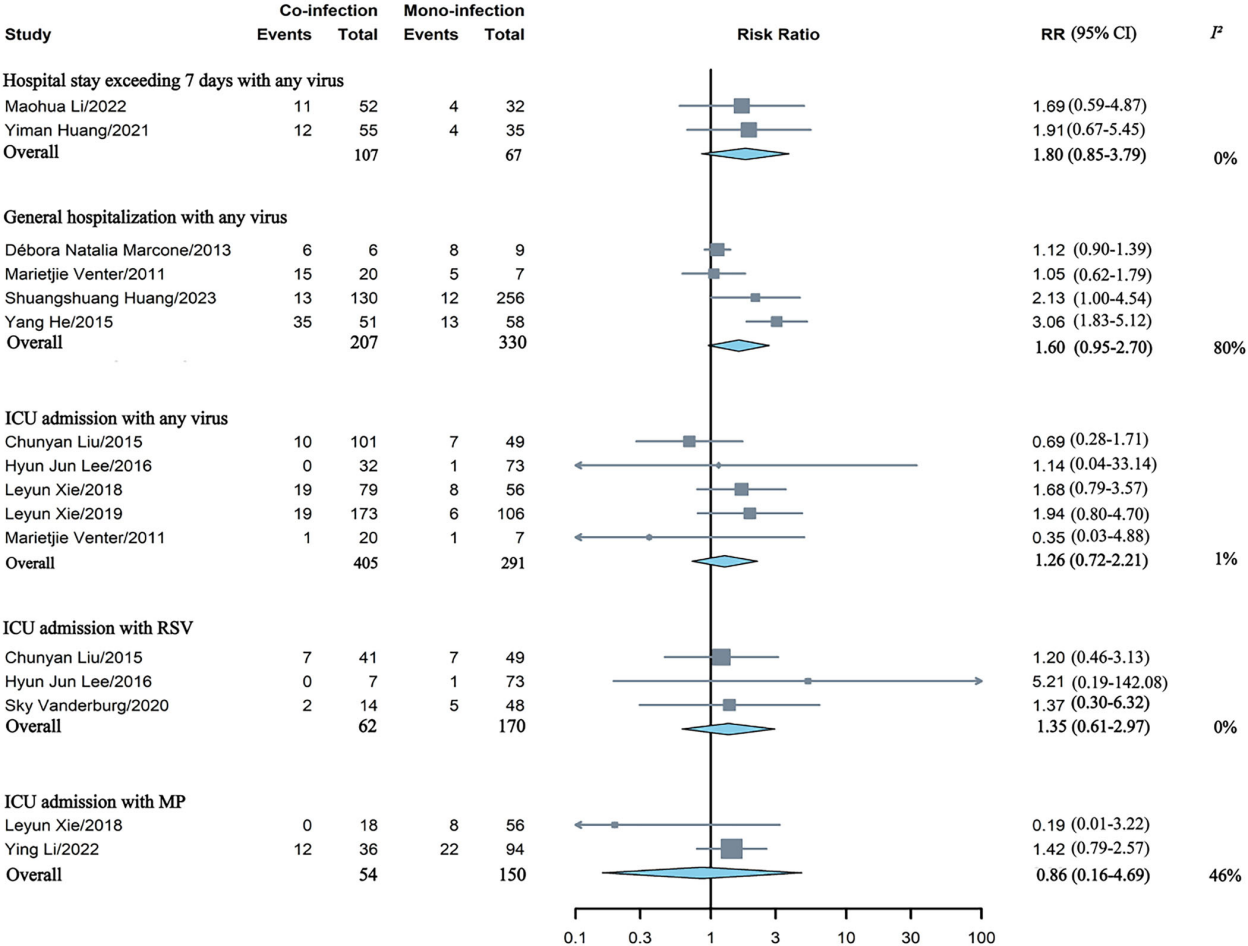
Comparative analysis of the risk of hospitalization in children with HAdV mono‐infection versus co‐infections involving any virus, RSV, or MP. ICU, intensive care unit; MP, *mycoplasma pneumoniae*; RR, risk ratios; RSV, respiratory syncytial virus.

**Figure 3:**
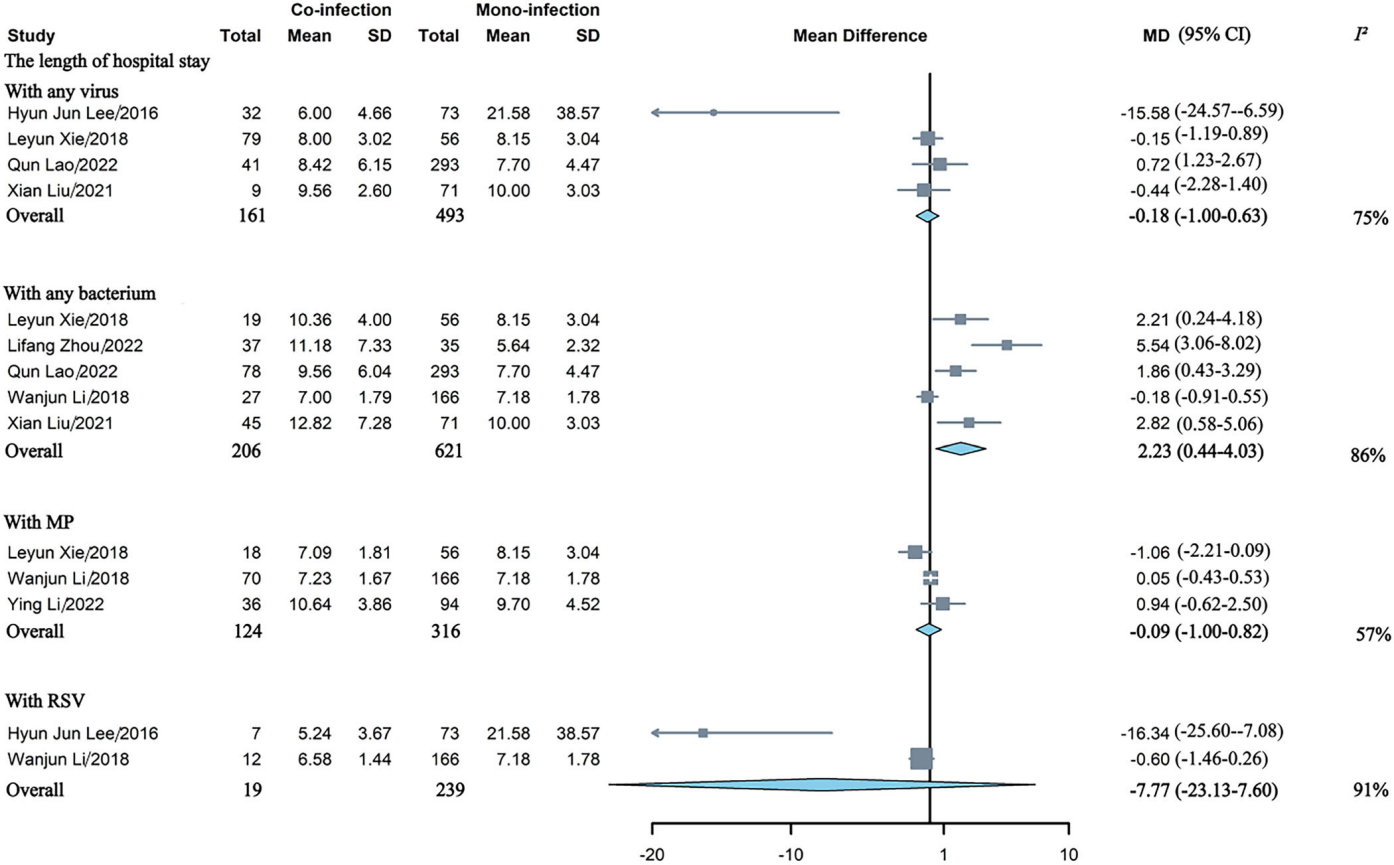
Comparative analysis of the length of hospital stay in children with HAdV mono‐ versus co‐infections involving any virus, RSV, or MP. MD, mean difference; MP, *mycoplasma pneumoniae*; RSV, respiratory syncytial virus.

Co‐infection of HAdV and any other virus was not linked to a higher risk of general hospitalization, and co‐infection of HAdV did not appear to elevate the risk of ICU admission for any virus, respiratory syncytial virus (RSV), or *mycoplasma pneumoniae* (MP) (Figure 2). Co‐infection of HAdV were found to be associated with prolonged hospital stay for any bacterium (MD = 2.23days, 95% CI: 0.44–4.03), which showed substantial heterogeneity (*I* ^*2*^ = 86%) (Figure 3).

### 3.2 The Association Between Co‐Infection and Pneumonia Severity

Nine studies documented common pneumonia risk among patients with HAdV mono‐infection compared to those with co‐infection involving any virus [14, 15, 18, 19, 20, 21, 22, 23, 24], and six studies compared the risk for co‐infection with bacterial pathogens [5, 15, 20, 23, 24, 25]. Sixteen studies documented the relationship of severe pneumonia with co‐infection involving any virus [1, 6, 14, 15, 18, 20, 21, 26, 27, 28, 29, 30, 31, 32], fourteen studies for co‐infection with any bacterium [5, 6, 15, 20, 25, 26, 27, 29, 30, 31, 33, 34, 35, 36], six studies for co‐infection with any fungus [26, 27, 28, 31, 34, 36], and two studies for co‐infection with any atypical pathogen [29, 30].

**Figure 4:**
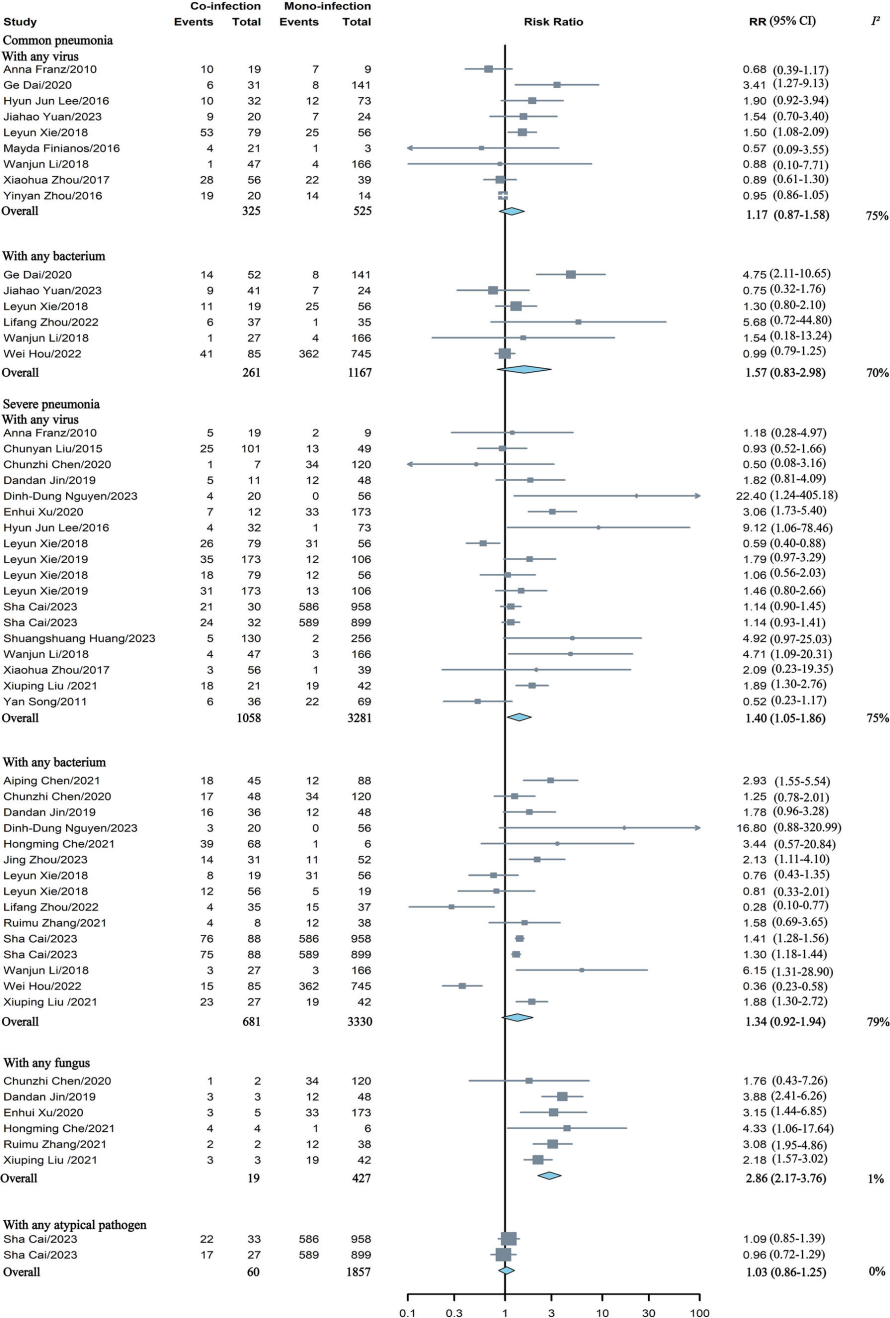
Comparative analysis of the risk of pneumonia severity in children with HAdV mono‐versus co‐infection involving any virus, bacterium, or fungus. BO, bronchiolitis obliterans; RR, risk ratios.

**Figure 5:**
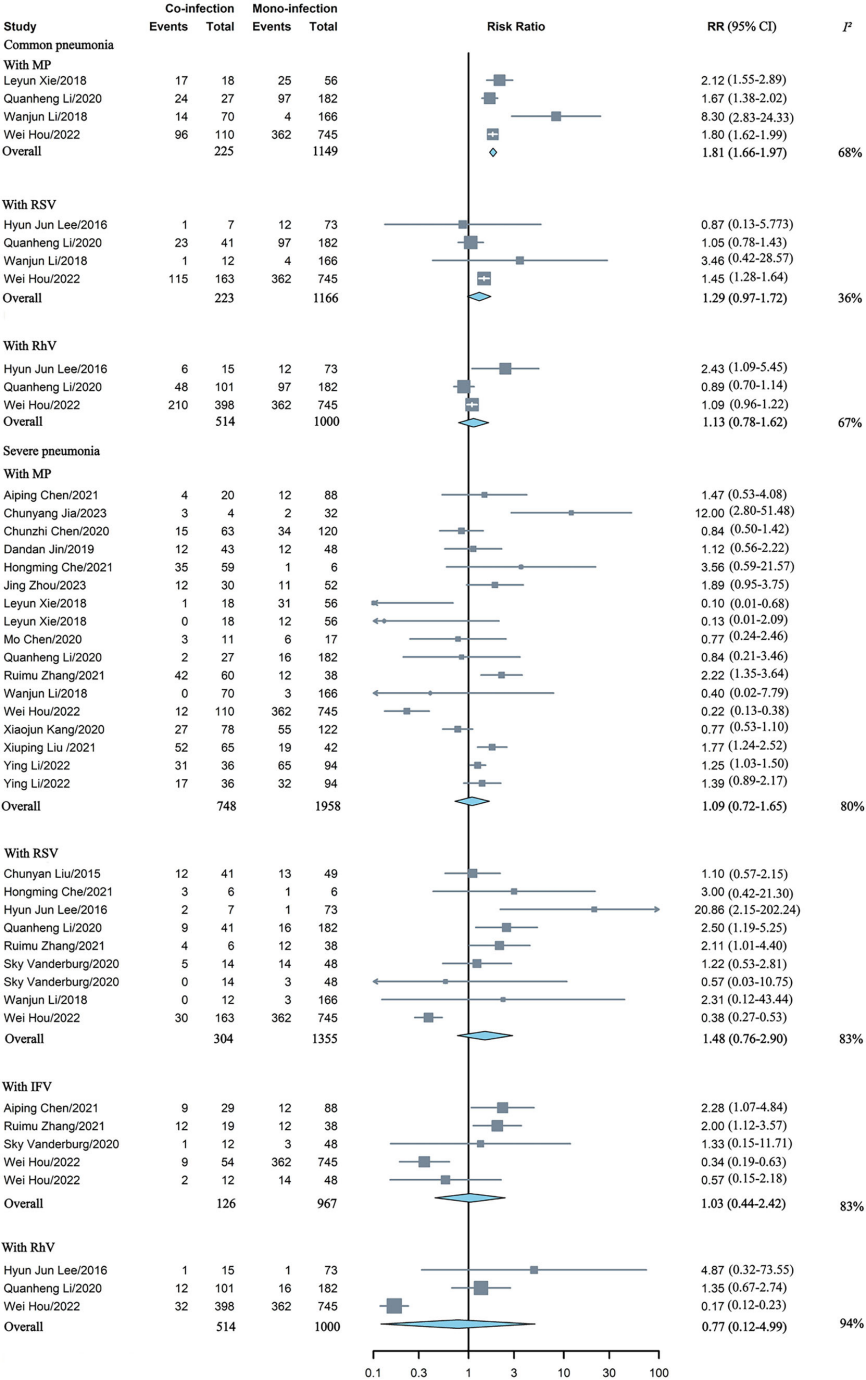
Comparative analysis of the risk of pneumonia severity among children with HAdV mono‐versus co‐infection involving single pathogen. IFV, influenza virus; MP, *mycoplasma pneumoniae*; RhV, rhinovirus; RR, risk ratios; RSV, respiratory syncytial virus.

Co‐infection was not linked to a higher risk of developing common pneumonia, whether for co‐infection with any virus or bacterium. Co‐infection involving HAdV was found to be associated with an increased risk of severe pneumonia when combined with any virus (RR = 1.40, 95% CI: 1.05–1.86) or fungus (RR = 2.86, 95% CI: 2.17–3.76), and these analyses showed high (*I* ^*2*^ = 75%) and low (*I* ^*2*^ = 1%) heterogeneity, respectively. However, co‐infection with any bacterium did not appear to elevate the risk of severe pneumonia (Figure 4). The meta‐analysis results regarding the severity of pneumonia in HAdV mono‐ versus co‐infection with a single pathogen were presented in Figure 5. Co‐infection of HAdV and MP elevated the risk of developing common pneumonia, with a RR of 1.81 (95% CI: 1.66–1.97), which showed high heterogeneity (*I* ^*2*^ = 68%).

### 3.3 The Association Between Co‐Infection With Risk of Deaths

**Figure 6:**
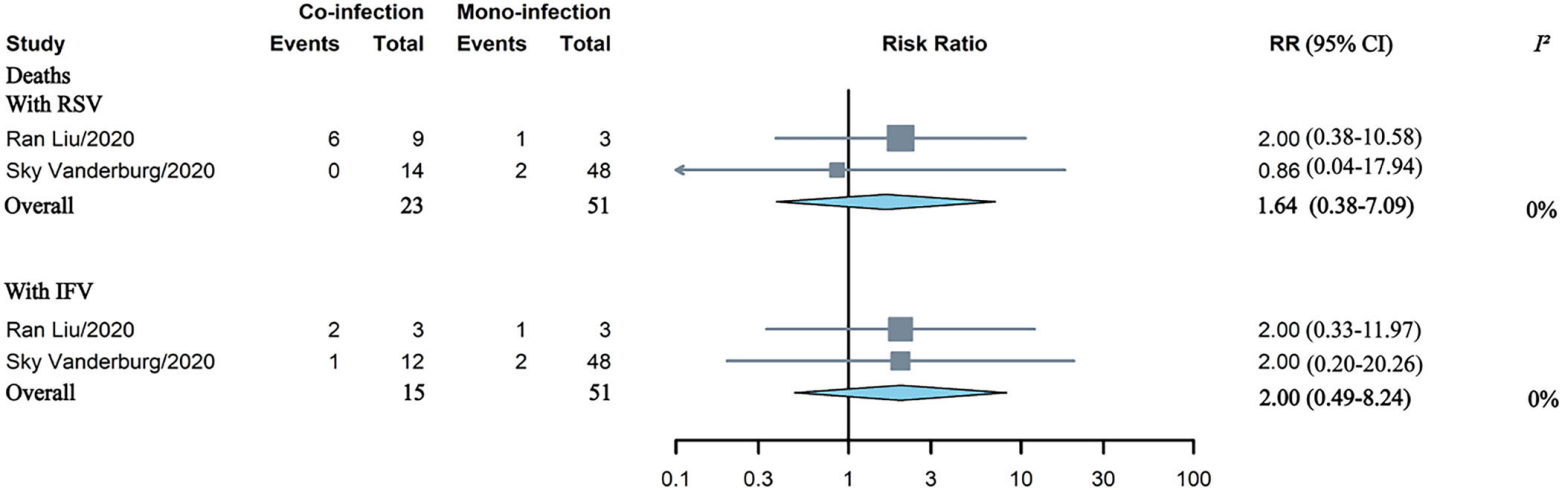
Comparative analysis of the risk of deaths in children with HAdV mono‐ versus co‐infection involving RSV and IFV. IFV, influenza virus; RR, risk ratios; RSV, respiratory syncytial virus.

Four studies documented death rates among patients with HAdV mono‐infection compared to those with co‐infection [14, 16, 22, 37]. Co‐infection with HAdV did not appear to elevate the risk of mortality associated with RSV (RR = 1.64, 95% CI: 0.38–7.09) or influenza virus (IFV) (RR = 2.00, 95% CI: 0.49–8.24) (Figure 6).

### 3.4 Publication Bias, and Sensitivity Analysis

Funnel plots illustrating the associations between hospitalization, pneumonia severity, and deaths outcomes with HAdV co‐infection were presented in Supporting Information S1: Figures S1–S5. These funnel plots were symmetric in qualitative assessment. In meta‐analyses comprising over two studies, Begg's and Egger's test similarly revealed no significant publication bias (Supporting Information S1: Table S6).

Supporting Information S1: Figures S6–S10 presented forest plots illustrating the correlations between hospitalization, pneumonia severity, deaths outcomes, and HAdV co‐infection. Sensitivity analysis suggested that the results of meta‐analyses were stable. In a meta‐analysis of the original study for severe pneumonia, twelve studies [1, 6, 15, 20, 21, 26, 27, 28, 29, 30, 31, 32] reported severe pneumonia among patients between HAdV mono‐ and co‐infection with any virus, and thirteen studies [5, 6, 15, 20, 26, 27, 29, 30, 31, 33, 34, 35, 36] with any bacterium, six studies [26, 27, 28, 31, 34, 36] with any fungus, and two studies [29, 30] with any atypical pathogen. Sensitivity analysis indicated that HAdV co‐infection was likely to be associated with increased risk of severe pneumonia with any bacterium (RR = 1.50, 95% CI: 1.05–2.16) and any fungus (RR = 2.86, 95% CI: 2.17–3.76), which showed high (*I* ^*2*^ = 78%) and low (*I* ^*2*^ = 1%) heterogeneity, respectively (Supporting Information S1: Figure S11).

## 4 Discussion

Our systematic review and meta‐analysis showed that compared with patients with HAdV single infection, co‐infection with other respiratory bacterial pathogens elevated the risk of prolonged hospitalization rather than any virus, RSV, or MP, co‐infection of HAdV and MP elevated the risk of common pneumonia, and co‐infection of HAdV and viral, bacterial, or fungal pathogens elevated the risk of severe pneumonia. Co‐infection of HAdV and viral pathogens was likely to increase the risk of severe pneumonia rather than RSV, IFV, RhV. Co‐infection of HAdV with RSV or IFV was unlikely to elevate the risk of death.

Co‐infection of HAdV appeared to elevate the risk of prolonged hospital stay due to bacterial presence rather than any virus, RSV, or MP. The study involving 310 patients in Korea also corroborated the link between co‐infection of HAdV and MP or other viruses and prolonged hospital stays [38], and the difference between the results of this study and our study may be related to the differences in the study area, sample size, categories of co‐infection pathogens, etc. This finding reflected differences in the interactions and effects of co‐infection by different pathogens. In HAdV co‐infection, when other viruses, RSV or MP interact with HAdV, they may be different from bacterial co‐infection in terms of pathological mechanism and immune response. Co‐infection of HAdV and bacteria with high risk of prolonged hospital stay might be three possible reasons. First, the possible unique molecular mechanism of the interaction between HAdV and bacteria made HAdV more likely to merge with bacteria and produced serious clinical consequences. Second, bacteria may exhibit resistance to standard antibiotics in cases of co‐infection, resulting in prolonged therapeutic interventions. Lastly, patients experiencing a combination of HAdV with bacterial infections may be more susceptible to secondary infections, such as empyema or pericarditis, and the management of these complications necessitated additional time and resources, thereby extending the duration of hospitalization. This finding showed substantial heterogeneity, which may be related to differences in health and medical care in the study area, pathogen detection methods, age range, and population susceptibility, etc. Prolonged hospital stay was linked to higher medical costs and a greater financial strain on families, and may also elevate the risk of hospital‐acquired infections. It is suggested that vigilance should be maintained for the presence of bacterial co‐infection in patients suffering from HAdV infections, to prevent the worsening of their condition.

Co‐infection of HAdV and MP appeared to elevate the risk of developing common pneumonia rather than hospitalization and severe pneumonia compared with patients with HAdV single infection, Other studies have come to similar conclusions, among which a study indicated that co‐infection of HAdV and MP was linked to a heightened risk of BO [39], and two studies indicated that co‐infection of HAdV and MP can result in more severe illness [40, 41]. Studies have shown that HAdV was the most common co‐infection in MP infection and was associated with RMPP [42]. The finding that HAdV infection with MP did not increase the risk of hospitalization was consistent with the conclusion of another study, which have shown that patients with co‐infection with HAdV have a longer duration of fever than patients with MP alone without longer hoapital stay [43]. Potential reasons could be associated with the regulation of inflammatory responses and injury and repair processes of lung tissue, and an exact biological explanation for the pathogenic impact of this particular pair of pathogens remained elusive.

Co‐infection of HAdV and viral or bacterial pathogens was likely to increase the risk of severe pneumonia. Specifically, HAdV combined with viruses led to an increased risk of severe pneumonia, the need for oxygen, and mechanical ventilation rather than bacteria, while HAdV combined with bacteria led to an increased risk of severe pneumonia rather than viruses. From this, we can speculate that co‐infection of HAdV and bacteria were more likely to cause severe pneumonia, and co‐infection of HAdV and viruses were more likely to increase oxygen supply and mechanical ventilation needs. Co‐infection of HAdV with bacteria was likely to increase the risk of severe pneumonia, which was consistent with the results of a systematic review published in 2022 [44]. Two possible reasons facilitated such viruses interactions, one of which involved the first virus enhancing the expression levels of host‐dependent factors in the infection of second virus. The second possible reason was that respiratory viruses can produce synergistic effects by causing cell fusion. This finding showed high heterogeneity, which may be related to differences in health and medical care in the study area, pathogen detection methods, age, and population susceptibility, etc. Competition interaction between viruses was also common typically through competition for nutrient resources, immune responses, or viral proteins [45], which aligned with the results of previous reviews on non‐HAdV‐specific viral co‐infection [4].

Co‐infection of HAdV and viral pathogens was likely to increase the risk of severe pneumonia rather than RSV, IFV, RhV, this finding suggested that there may be other virus co‐infection with HAdV leading to severe pneumonia. The study involving 111 patients indicated that co‐infection of HAdV and cytomegalovirus was linked to a heightened risk of severe pneumonia [46]. Furthermore, it has been reported that co‐infection of HAdV and RSV was associated with an elevated risk of BO, and co‐infection of HAdV and measles virus was associated with an elevated risk of mortality [47]. We found co‐infection of HAdV and RSV was unlikely to increase the risk of severe pneumonia, it was supposed that the co‐infection of HAdV and RSV can only cause common pneumonia rather than severe pneumonia, which was similar to the results of co‐infection of HAdV and MP.

Co‐infection of HAdV with fungi was also likely to increase the risk of severe pneumonia. This finding was consistent with the results of a systematic review published in 2022 [44]. Three potential reasons could elucidate this discovery. Initially, viral infections, including HAdV, can inflict harm and necrosis upon respiratory epithelial cells, thereby compromising the respiratory tract's physical barrier and facilitating easier penetration and infection of underlying tissues by fungi [48]. Secondly, HAdV infection might provoke an immunosuppressive reaction, which diminished the host's resistance to fungal infections. Thirdly, the combination of HAdV with fungal infections could precipitate an overzealous inflammatory response, leading to substantial infiltration of inflammatory cells and cytokine release. This intense inflammatory reaction could further deteriorate lung tissue and heighten the risk of severe pneumonia. Co‐infection of HAdV with RSV or IFV was unlikely to elevate the risk of death, which was consistent with the results from two studies [14, 22]. A plausible explanation may be due to competitive dynamics among viruses; another possible reason was that when multiple pathogens infect an individual simultaneously, there may be cross‐reactivity among these targeted immune responses, which could bolster the host's defense against various pathogens. Variations in patients' age, immune status, and pre‐existing conditions significantly influenced the progression and outcome of the disease. Nevertheless, based on the point estimates in this study, it can be seen that HAdV co‐infection increased the risk of death, which may be attributed to the wide CI caused by the small number of included studies. A more definitive conclusion needed confirmed by a larger number of studies.

### 4.1 Limitations

This meta‐analysis has several limitations that need to be considered when interpreting the results. First, co‐infection of HAdV and other pathogens in our study did not inherently suggest a pathogenic effect from either agent. It is also possibe that the increased risk of clinical outcomes in patients may not be attributed to the co‐infection of two pathogens, but rather to the pathogenicity of the pathogen with which HAdV was coinfected. A study indicated that individuals infected with both HAdV and MP necessitated a more extended hospitalization period and a greater reliance on oxygen supply compared to those with MP alone. To some extent, we can deduce that the heightened risk of common pneumonia associated with HAdV co‐infection alongside MP in this study was more likely attributable to the synergistic effect between the two pathogens. Second, several meta‐analyses indicated high heterogeneity, and subgroup or meta‐regression analyses were not feasible to elucidate the origins of this variation due to limited studies. Third, this study did not account for the impact of HAdV co‐infection with multiple pathogens on clinical outcomes due to insufficient relevant data. Fourthly, the estimation of between‐study heterogeneity was prone to bias towards zero when the number of studies included was small in a random‐effects meta‐analysis, rendering the pooled estimate more susceptible to outliers.

## 5 Conclusions

Our research indicated that co‐infection of HAdV and viral pathogens elevated the risk of severe pneumonia rather than hospitalization and commom pneumonia, co‐infection of HAdV and bacterial pathogens elevated the risk of hospitalization and commom pneumonia rather than severe pneumonia, co‐infection of HAdV and fungal pathogens elevated the risk of severe pneumonia, and co‐infection of HAdV and MP elevated the risk of commom pneumonia rather than hospitalization and severe pneumonia among pediatric patients. These co‐infections undoubtedly led to increased consumption of medical resources, and it is implied that the ecological and biological mechanisms underlying potential interactions between HAdV and other microorganisms warranted further study.

## Author Contributions

**Dandan Niu:** investigation, resources, methodology, formal analysis, writing – original draft, and editing. **Yanxiao Gao:** investigation, resources, writing – original draft, funding acquisition, and editing. **Yingluan Zhang:** investigation, resources, writing – original draft, and editing. **Qiuying Lv:** investigation, writing – review. **Yiwen Jiang:** investigation, writing – review, and editing. **Yuanxi Jia:** investigation, writing – review, and editing. **Zhigao Chen:** investigation, writing – review. **Honglin Wang:** investigation, writing – review. **Yanpeng Cheng:** investigation, writing – review. **Feng Sha:** investigation, writing – review. **Meng Ren:** investigation, writing – review. **Yixiong Chen:** investigation, writing – review. **Xindong Zhang:** investigation, writing – review. **Zhen Zhang:** investigation, conceptualization, writing – review, supervision. **Jinling Tang:** investigation, conceptualization, and funding acquisition, writing – review, supervision. **Tiejian Feng:** investigation, conceptualization, funding acquisition, writing – review, supervision.

## Ethics Statement

This study was approved by the Science and Technology Ethics Review Committee of Shenzhen Center for Disease Control and Prevention. This study involves clinical‐epidemiological data of the patients from the published articles, so the informed consent signed by the patients was unnecessary.

## Conflicts of Interest

The authors declare no conflicts of interest.

## Supporting information

Supplementary file‐clean.

## Data Availability

The data that supports the findings of this study are available in the Supporting material of this article.
