# Supplementary material for: Systematic Review and Meta‐Analysis of the Association Between Clinical Severity and Co‐Infection of Human Adenovirus With Other Respiratory Pathogens in Children
Source: J Med Virol. 2025 Apr 29;97(5):e70370. doi: 10.1002/jmv.70370 (PMC12038779; doi:10.1002/jmv.70370)
Supplement: Supplementary file 1 — Supplementary file‐clean. [file JMV-97-e70370-s001.doc]

# Supplementary Materials

**Title**: **Systematic review and meta-analysis of the association between clinical severity and co-infection of human adenovirus with other respiratory pathogens in children**

**Short title:** **the clinical severity and co-infection of human adenovirus**

**Authors**: Dandan Niu^1, 2, 3, #^|Yanxiao Gao^1, #^ | Yingluan Zhang^2, 3, #^| Qiuying Lv^2, 3^| Yiwen Jiang^1^| Yuanxi Jia^1^| Zhigao Chen^2, 3^| Honglin Wang^2, 3^| Yanpeng Cheng^2, 3^| Feng Sha^1^| Meng Ren^4^| Yixiong Chen^4^| Xindong Zhang^4^| Zhen Zhang^2, 3,*^| Jinling Tang^1,5,*^| Tiejian Feng^2, 3,*^

**Current affiliations**:

^1^Shenzhen Institute of Advanced Technology, Chinese Academy of Sciences, Shenzhen, Guangdong Province, China

^2^Department of Communicable Diseases Control and Prevention, Shenzhen Center for Disease Control and Prevention, Shenzhen, Guangdong Province, China

^3^Shenzhen Research Center for Communicable Disease Control and Prevention, Chinese Academy of Medical Sciences, Shenzhen, Guangdong Province, China

^4^Department of Communicable Diseases Control and Prevention, Baoan District Center for Disease Control and Prevention, Shenzhen, Guangdong Province, China

^5^Faculty of Computer Science and Control Engineering, Shenzhen University of Advanced Technology, Shenzhen, Guangdong Province, China

**Supplementary Table 1.** Preferred Reporting Items for Systematic Reviews and Meta-analyses (PRISMA) checklist**(1)**

| **Section and Topic** | **#** | **Checklist item** | **Reported in main text on** |
| --- | --- | --- | --- |
| **Title** | | |  |
| Title | 1 | Identify the report as a systematic review, meta-analysis, or both. | p.1 |
| **Abstract** | | |  |
| Structured summary | 2 | Provide a structured summary including, as applicable: background; objectives; data sources; study eligibility criteria, participants, and interventions; study appraisal and synthesis methods; results; limitations; conclusions and implications of key findings; systematic review registration number. | p.2 |
| **Introduction** | | |  |
| Rationale | 3 | Describe the rationale for the review in the context of existing knowledge. | p.3 |
| Objectives | 4 | Provide an explicit statement of the objective(s) or question(s) the review addresses. | p.3-4 |
| **Methods** | | |  |
| Protocol and registration | 5 | Indicate if a review protocol exists, if and where it can be accessed (e.g., Web address), and, if available, provide registration information including registration number. | Registered in PROSEPRO with CRD42024547528 |
| Eligibility criteria | 6 | Specify study characteristics (e.g., PICOS, length of follow-up) and report characteristics (e.g., years considered, language, publication status) used as criteria for eligibility, giving rationale. | p.4-5 |
| Information sources | 7 | Describe all information sources (e.g., databases with dates of coverage, contact with study authors to identify additional studies) in the search and date last searched. | p.4 |
| Search | 8 | Present full electronic search strategy for at least one database, including any limits used, such that it could be repeated. | Box S1 |
| Study Selection | 9 | State the process for selecting studies (i.e., screening, eligibility, included in systematic review, and, if applicable, included in the meta-analysis). | p.4-5 |
| Data collection process | 10 | Describe method of data extraction from reports (e.g., piloted forms, independently, in duplicate) and any processes for obtaining and confirming data from investigators. | p.4-5 |
| Data items | 11 | List and define all outcomes for which data were sought. Specify whether all results that were compatible with each outcome domain in each study were sought (e.g. for all measures, time points, analyses), and if not, the methods used to decide which results to collect. | p.4-5 |
| Risk of bias in individual studies | 12 | Describe methods used for assessing risk of bias of individual studies (including specification of whether this was done at the study or outcome level), and how this information is to be used in any data synthesis. | p.5-6 |
| Summary measures | 13 | State the principal summary measures (e.g., risk ratio, difference in means). | p.5-6 |
| Synthesis of results | 14 | Describe the methods of handling data and combining results of studies, if done, including measures of consistency (e.g., I^2^ ) for each meta-analysis. | p.6 |
| Risk of bias across studies | 15 | Specify any assessment of risk of bias that may affect the cumulative evidence (e.g., publication bias, selective reporting within studies). | p.6 |
| Additional analyses | 16 | Describe methods of additional analyses (e.g., sensitivity or subgroup analyses, meta-regression), if done, indicating which were pre-specified. | p.6 |
| **Results** | | |  |
| Study selection | 17 | Give numbers of studies screened, assessed for eligibility, and included in the review, with reasons for exclusions at each stage, ideally with a flow diagram. | p.6; Figure 1 |
| Study characteristics | 18 | For each study, present characteristics for which data were extracted (e.g., study size, PICOS, follow-up period) and provide the citations. | Table S2-3 |
| Risk of bias | 19 | Present data on risk of bias of each study and, if available, any outcome level assessment (see item 12). | Table S4 |
| Results of individual studies | 20 | For all outcomes considered (benefits or harms), present, for each study: (a) simple summary data for each intervention group (b) effect estimates and confidence intervals, ideally with a forest plot. | p.6-8; Figure 2-6 |
| Synthesis of results | 21 | Present results of each meta-analysis done, including confidence intervals and measures of consistency. | p.6-8; Figure 2-6; Table S5 |
| Risk of bias | 22 | Present results of any assessment of risk of bias across studies (see Item 15). | Table S4 |
| Additional analysis | 23 | Give results of additional analyses, if done (e.g., sensitivity or subgroup analyses, meta-regression [see Item 16]). | p.8; Figure S1-11, Table S6 |
| **Discussion** | | |  |
| Summary of evidence | 24 | Summarize the main findings including the strength of evidence for each main outcome; consider their relevance to key groups (e.g., healthcare providers, users, and policy makers). | p.8-11 |
| Limitations | 25 | Discuss limitations at study and outcome level (e.g., risk of bias), and at review-level (e.g., incomplete retrieval of identified research, reporting bias). | p.11-12 |
| Conclusions | 26 | Provide a general interpretation of the results in the context of other evidence, and implications for future research. | p.12 |
| **Funding** | | |  |
| Funding | 27 | Describe sources of funding for the systematic review and other support (e.g., supply of data); role of funders for the systematic review. | p.13 |

Abbreviations: p = Page(s).

**Supplementary Box 1.** Data sources and search criteria for a systematic review of the association between clinical outcomes and HAdV co-infection among children

**MEDLINE: (227 Results)**

#1: "adenovirus infections, human"[MeSH Terms] OR "infections human adenovirus"[All Fields] OR "Human Adenovirus Infections"[All Fields] OR ("adenovirus infections, human"[MeSH Terms] OR ("adenovirus"[All Fields] AND "infections"[All Fields] AND "human"[All Fields]) OR "Human Adenovirus Infections"[All Fields] OR ("adenovirus"[All Fields] AND "infection"[All Fields] AND "human"[All Fields])) OR "Human Adenovirus Infection"[All Fields] OR "infection human adenovirus"[All Fields] OR "pharyngo conjunctival fever"[All Fields] OR ("adenovirus infections, human"[MeSH Terms] OR ("adenovirus"[All Fields] AND "infections"[All Fields] AND "human"[All Fields]) OR "Human Adenovirus Infections"[All Fields] OR ("fever"[All Fields] AND "pharyngo"[All Fields] AND "conjunctival"[All Fields])) OR "pharyngo conjunctival fever"[All Fields]

#2: "Coinfection"[MeSH Terms] OR "Coinfections"[All Fields] OR "Coinfections"[All Fields] OR "Mixed Infection"[All Fields] OR "infection mixed"[All Fields] OR "infections mixed"[All Fields] OR "Mixed Infections"[All Fields] OR "Polymicrobial Infection"[All Fields] OR "infection polymicrobial"[All Fields] OR "infections polymicrobial"[All Fields] OR "Polymicrobial Infections"[All Fields] OR "co infection"[All Fields] OR "co infection"[All Fields] OR "Co-infection"[All Fields] OR "Secondary Infection"[All Fields] OR "infection secondary"[All Fields] OR "infections secondary"[All Fields] OR "Secondary Infection"[All Fields]

#3: "Superinfection"[MeSH Terms] OR "Superinfections"[All Fields] OR "Microbial Superinvasion"[All Fields]

#4: #2 or #3

#5: "Death"[MeSH Terms] OR "end of life"[All Fields] OR "end of life"[All Fields] OR "Determination of Death"[All Fields] OR "Near-Death Experience"[All Fields] OR "Cardiac Death"[All Fields] OR "death cardiac"[All Fields]

#6: "Bronchiolitis obliterans"[MeSH Terms] OR "Bronchiolitis obliterans"[All Fields]

#7: ("Mortality"[MeSH Terms] AND "Child Mortality"[MeSH Terms]) OR "Mortalities"[All Fields] OR "Mortality Rate"[All Fields] OR "Mortality Rates"[All Fields] OR "rate mortality"[All Fields] OR "Death Rate"[All Fields] OR "Death Rates"[All Fields] OR "rate death"[All Fields] OR "Case Fatality Rate"[All Fields] OR "Case Fatality Rates"[All Fields] OR "rate case fatality"[All Fields] OR "rates case fatality"[All Fields] OR "CFR Case Fatality Rate"[All Fields] OR "Crude Death Rate"[All Fields] OR "Crude Death Rates"[All Fields] OR "death rate crude"[All Fields] OR "rate crude death"[All Fields] OR "Crude Mortality Rate"[All Fields] OR "Crude Mortality Rates"[All Fields] OR "mortality rate crude"[All Fields] OR "rate crude mortality"[All Fields] OR "decline mortality"[All Fields] OR "Mortality Declines"[All Fields] OR "Mortality Decline"[All Fields] OR "Mortality Determinants"[All Fields] OR "determinants mortality"[All Fields] OR "determinant mortality"[All Fields] OR "Mortality Determinant"[All Fields] OR "mortality differential"[All Fields] OR "Differential Mortality"[All Fields] OR "Differential Mortalities"[All Fields] OR "age specific death rate"[All Fields] OR "Age-Specific Death Rates"[All Fields] OR "death rate age specific"[All Fields] OR "rate age specific death"[All Fields] OR "age specific death rate"[All Fields] OR "mortality excess"[All Fields] OR "Excess Mortality"[All Fields] OR "Excess Mortalities"[All Fields]

#8: "Hospitalization"[MeSH Terms] OR "Hospitalizations"[All Fields]

#9: "ICU admission"[MeSH Terms] OR "ICU admission"[All Fields]

#10: "Oxygen Inhalation Therapy"[MeSH Terms] OR "Oxygen Inhalation Therapies"[All Fields] OR "therapy oxygen inhalation"[All Fields]

#11: "Respiration, Artificial"[MeSH Terms] OR "Artificial Respiration"[All Fields] OR "Artificial Respirations"[All Fields] OR "Respirations Artificial"[All Fields] OR "Ventilation Mechanical"[All Fields] OR "Mechanical Ventilations"[All Fields] OR "Ventilations Mechanical"[All Fields] OR "Mechanical Ventilation"[All Fields]

#12: "Pneumonia"[MeSH Terms] OR "Pneumonias"[All Fields] OR "Lobar Pneumonia"[All Fields] OR "Lobar Pneumonias"[All Fields] OR "pneumonia lobar"[All Fields] OR "Experimental Lung Inflammation"[All Fields] OR "lung inflammation experimental"[All Fields] OR "Pneumonitis"[All Fields] OR "Pneumonitides"[All Fields] OR "Pulmonary Inflammation"[All Fields] OR "inflammation pulmonary"[All Fields] OR "inflammations pulmonary"[All Fields] OR "Pulmonary Inflammations"[All Fields] OR "Lung Inflammation"[All Fields] OR "inflammation lung"[All Fields] OR "inflammations lung"[All Fields] OR "Lung Inflammations"[All Fields]

#13: #5 OR #6 OR #7 OR #8 OR #9 OR #10 OR #11 OR #12

#14: #1 AND #4 AND #13

**Embase: (186 Results)**

#1: 'human adenovirus'/exp OR 'human adenovirus' OR 'human adenovirus infection'/exp OR 'human adenovirus infection'

#2: 'coinfection'/exp OR 'coinfection' OR 'mixed infection'/exp OR 'mixed infection' OR 'superinfection'/exp OR 'superinfection'

#3: 'mortality'/exp OR 'mortality' OR 'death'/exp OR 'death' OR 'icu admission'/exp OR 'icu admission' OR 'hospitalization'/exp OR 'hospitalization' OR 'oxygen inhalation therapy'/exp OR 'oxygen inhalation therapy' OR 'pneumonia'/exp OR 'pneumonia' OR 'severe pneumonia'/exp OR 'severe pneumonia' OR 'mechanical ventilations'/exp OR 'mechanical ventilations' OR 'bronchiolitis obliterans'/exp OR 'bronchiolitis obliterans'

#4: #1 AND #2 AND #3

**Web of Science: (509 Results)**

#1: TS=(human adenovirus) OR TS=(human adenovirus infection)

#2: (TS=(coinfection) OR TS=(mixed infection) OR TS=(superinfection)

#3: TS=(mortality) OR TS=(death) OR TS=(icu admission) OR TS=(hospitalization) OR TS=(oxygen inhalation therapy) OR TS=(pneumonia) OR TS=(severe pneumonia) OR TS=(mechanical ventilations) OR TS=(bronchiolitis obliterans)

#4: #1 AND #2 AND #3

**China National Knowledge Infrastructure: (275 Results)**

#1: TI=human adenovirus OR TI=human adenovirus infection

#2: TI=coinfection OR TI=mixed infection OR TI=superinfection

#3: TI=pneumonia OR TI=severe peneumonia OR TI=hospitalization OR TI=death OR TI=icu admission OR TI=oxygen inhalation therapy OR TI=mechanical ventilations OR TI=bronchiolitis obliterans

#4: #1 AND #2 AND #3

**Wanfang data: (400 Results)**

#1: TI:(human adenovirus) OR TI:(human adenovirus infection)

#2: TI:(coinfection) OR TI:(mixed infection) OR TI:(superinfection)

#3: TI:(death) OR TI:(hospitalization) OR TI:(pneumonia) OR TI:(severe pneumonia) OR TI:(icu admission) OR TI:(oxygen inhalation therapy) OR TI:(mechanical ventilations) OR TI:(bronchiolitis obliterans)

#4: #1 AND #2 AND #3

**Chongqing VIP information: (851 Results)**

#1: U=human adenovirus OR U=human adenovirus infection OR U=adenovirus, human

#2: U=coinfection OR U=mixed infection OR U=superinfection

#3: U=mechanical ventilations OR U=oxygen inhalation therapy OR U=icu admission OR U=death OR OR U=pneumonia OR U=severe pneumonia OR U=bronchiolitis obliterans

#4: #1 AND #2 AND #3

**Total:** 2448 Results by May 16th, 2024

**Supplementary Table 2.** Studies reporting three clinical outcomes among children with HAdV mono- versus co-infection

| **Author,**  **year of publication** | **Year(s) of**  **data**  **collection** | **Country** | **Study site** | **Diagnostic methods** | **Age** | **Clinical diagnosis** | **Sample size** | **Hospitalization** | **Pneumonia severity** | **Deaths** |
| --- | --- | --- | --- | --- | --- | --- | --- | --- | --- | --- |
| Aiping Chen, 2021(2) | 2018-19 | China | IP | PCR | ≤6y | CAP | 117 | No | Yes | No |
| Anna Franz, 2010(3) | 2006-08 | Germany | IP | PCR | <16y | ALRI | 28 | No | Yes | No |
| Caiyun Wang, 2021(4) | 2018-19 | China | IP | PCR | 28d-18y | ARI | 488 | Yes | Yes | No |
| Caiyun Wang, 2021(5) | 2018-19 | China | IP | DFA | 39d-12y | RTD | 488 | Yes | Yes | No |
| Chunyan Liu, 2015(6) | 2007-12 | China | OP/IP/ER | PCR | 0.5m-19y | ARI | 150 | Yes | Yes | No |
| Chunyang Jia, 2023(7) | 2019-21 | China | IP | EIA/Culture | <12y | CAP | 38 | No | Yes | No |
| Chunzhi Chen, 2020(8) | 2019 | China | IP | PCR/EIA/Culture | 17m-4y | CAP | 157 | No | Yes | No |
| Dandan Jin, 2019(9) | 2015-17 | China | OP/IP | PCR/Culture | 28d-14y | CAP | 118 | No | Yes | No |
| Daoxiang Rong, 2020(10) | 2017-19 | China | IP | DFA/EIA/Culture | 6m-6y | CAP | 120 | No | Yes | No |
| Débora Natalia Marcone, 2013(11) | 2008-10 | Argentina | IP/ER | DFA/IFA/PCR | <6y | RTD | 15 | Yes | No | No |
| Dinh-Dung Nguyen, 2023(12) | 2022 | Vietnam | OP/IP | PCR | 2.7(2.4,3)y | ARI | 91 | No | Yes | No |
| Enhui Xu, 2020(13) | 2019 | China | IP | DFA/PCR/EIA/Culture | <10y | CAP | 178 | No | Yes | No |
| Fuliang Jiang, 2022(14) | 2019 | China | IP | PCR | ≤12y | ARI | 63 | Yes | Yes | No |
| Ge Dai, 2020(15) | 2011-17 | China | IP | DFA/EIA/Culture | 1m-69m | CAP | 172 | No | Yes | No |
| Hongming Che, 2021(16) | 2018-21 | China | IP | PCR/EIA | 1m-9y | CAP | 80 | No | Yes | No |
| Hongwei Chen, 2022(17) | 2019 | China | IP | PCR/DFA | <10y | ARI | 1327 | Yes | Yes | No |
| Hyun Jun Lee, 2016(18) | 2012-14 | Korea | OP/IP | PCR | <20y | RTD | 105 | Yes | Yes | Yes |
| Jiahao Yuan, 2023(19) | 2019-20 | China | IP | PCR/EIA | 1m-14y | CAP | 112 | No | Yes | No |
| Jiaotian Huang , 2021(20) | 2018-19 | China | IP | DFA | 42d-8y | SP | 114 | No | Yes | Yes |
| Jie Lin, 2020(21) | 2016-17 | China | IP | PCR | 2m-12y | RTD | 84 | Yes | Yes | Yes |
| Jing Zhou, 2023(22) | 2019 | China | IP | PCR/DFA/EIA | 1m-14y | CAP | 97 | No | Yes | No |
| Leyun Xie, 2018(23) | 2011-14 | China | IP | PCR | 1m-13y | CAP | 174 | Yes | Yes | No |
| Leyun Xie, 2019(24) | 2007-14 | China | IP | PCR | 1d-14y | ALRI | 279 | Yes | Yes | No |
| Lifang Zhou, 2022(25) | 2018-19 | China | IP | DFA/Culture | 1m-14y | SP | 72 | Yes | Yes | Yes |
| Lingjian Zeng, 2021(26) | 2012-19 | China | IP | EIA | 10m-16y | ALRI | 10 | No | Yes | Yes |
| Lu Xu, 2021(27) | 2018-19 | China | IP | PCR/DFA | 4m-11y | CAP | 111 | No | Yes | No |
| Maohua Li, 2022(28) | 2016-18 | China | OP/IP | PCR | <14y | CAP | 84 | Yes | No | No |
| Marietjie Venter, 2011(29) | 2006-07 | South Africa | OP/IP | PCR | <5y | ARI | 27 | Yes | No | No |
| Mayda Finianos, 2016(30) | 2013-14 | Lebanon | IP | PCR | ≤16y | ARI | 24 | No | Yes | No |
| Miri Dotan, 2022(31) | 2005-20 | Israel | IP | PCR/DFA/EIA | <18y | RTD | 207 | Yes | No | No |
| Mo Chen, 2020(32) | 2019 | China | IP | PCR/EIA/Culture | 1y-9y | CAP | 28 | No | Yes | No |
| P.Q. Huang, 2022(33) | 2018-19 | China | IP | PCR/Culture | 0.2y-7.8y | RTD | 143 | Yes | No | Yes |
| Patricia Murtagh, 2009(34) | 1988-2005 | Argentina | IP | IFA | <6y | ALRI | 415 | No | No | Yes |
| Peipei Du, 2020(35) | 2016-19 | China | IP | DFA/PCR/Culture | 2m-13y | CAP | 576 | No | Yes | No |
| Qian Hu , 2021(36) | 2017-19 | China | IP | PCR | 1m-16y | CAP | 541 | No | Yes | No |
| Qing Lou, 2021(37) | 2019 | China | IP | DFA/PCR | 1m-5.5y | CAP | 168 | No | Yes | No |
| Quanheng Li, 2020(38) | 2016-18 | China | IP | PCR | 1m-9y | ARI | 476 | No | Yes | No |
| Qun Lao, 2022(39) | 2019 | China | IP | PCR/EIA | <14y | RTD | 454 | Yes | No | No |
| Ran Liu, 2020(40) | 2017-19 | China | IP | DFA/Culture | 1m-12y | SP | 12 | No | No | Yes |
| Ruimu Zhang, 2021(41) | 2019 | China | IP | PCR/DFA | <10y | CAP | 111 | No | Yes | No |
| Sha Cai, 2023(42) | 2010-19 | China | IP | DFA | <10y | CAP | 1046 | No | Yes | No |
| Sha Cai, 2023(43) | 2010-19 | China | IP | PCR/DFA/EIA | ≤14y | CAP | 1046 | No | Yes | No |
| Shuangshuang Huang, 2023(44) | 2020-21 | China | OP/IP | PCR | 2d-11.5y | ARI | 386 | Yes | Yes | No |
| Silan Liu, 2021(45) | 2019 | China | IP | PCR/DFA/Culture | <14y | CAP | 143 | No | Yes | No |
| Sky Vanderburg, 2020(46) | 2018 | China | IP | PCR | <5y | RTD | 60 | Yes | Yes | Yes |
| Wanjun Li, 2018(47) | 2016-17 | China | IP | DFA/EIA/Culture | 2m-14y | ARI | 213 | Yes | Yes | No |
| Wei Hou, 2022(48) | 2017-21 | China | IP | PCR | 1m-18y | RTD | 2356 | No | Yes | No |
| Xian Liu, 2021(49) | 2018-19 | China | IP | PCR/DFA | 2m-97m | SP | 149 | Yes | No | No |
| Xiaohua Zhou, 2017(50) | 2012-16 | China | IP | PCR | <18y | RTD | 95 | No | Yes | No |
| Xiaojun Kang, 2020(51) | 2019 | China | IP | DFA/EIA | <10y | CAP | 200 | No | Yes | No |
| Xiaoling Li, 2021(52) | 2018-19 | China | IP | PCR/DFA/EIA/Culture | <10y | CAP | 127 | No | Yes | No |
| Xiaoyan Sun, 2022(53) | 2019-21 | China | IP | PCR/DFA/EIA | <18y | CAP | 128 | No | Yes | No |
| Xinfen Yu, 2018(54) | 2011-13 | China | OP/IP | PCR | 1m-10y | RTD | 71 | Yes | No | No |
| Xiuping Liu, 2021(55) | 2019 | China | IP | DFA/EIA | <10y | CAP | 131 | No | Yes | No |
| Xuan Zheng, 2022(56) | 2020-21 | China | IP | PCR | <18y | RTD | 410 | Yes | Yes | No |
| Xueping Xu, 2020(57) | 2017-19 | China | IP | DFA/EIA/PCR | 28d-14y | SP | 125 | No | Yes | No |
| Yan Hong, 2021(58) | 2019 | China | IP | PCR/Culture | 29d-14y | CAP | 133 | No | Yes | No |
| Yan Song, 2011(59) | 2009-11 | China | IP | PCR | 1m-12y | RTD | 105 | No | Yes | No |
| Yang He, 2015(60) | 2011-12 | China | OP/IP | PCR | <18y | ARI | 109 | Yes | No | No |
| Yanling Chen, 2021(61) | 2019 | China | IP | PCR/Culture | 28d-14y | CAP | 60 | No | Yes | No |
| Yi Chen, 2014(62) | 2012-13 | China | IP | PCR | 1m-13y | CAP | 78 | No | Yes | No |
| Yiman Huang, 2021(63) | 2018-19 | China | IP | PCR | <14y | RTD | 90 | Yes | No | No |
| Ying Li, 2022(64) | 2018-19 | China | IP | PCR/DFA/EIA | <10y | CAP | 130 | Yes | Yes | No |
| Yinyan Zhou, 2016(65) | 2011-13 | China | OP/IP | PCR | 3m-14y | ARI | 104 | No | Yes | Yes |
| Yuanmei Huang, 2017(66) | 2011-15 | China | IP | DFA/EIA/PCR | 1m-14y | RTD | 392 | Yes | Yes | No |

Abbreviations: IP=inpatient, OP=outpatients, ER=emergency room, PCR=polymerase chain reaction, DFA=direct immunofluorescence assay, IFA=indirect immunofluorescence assay, EIA=enzyme-linked immunosorbent assay, ALRI=acute lower respiratory infection, ARI=acute respiratory infection, RTD=respiratory tract disease, CAP=community acquired pneumonia, SP=severe pneumonia, ICU=intensive care unit, m=month, y=year.

**Supplementary Table 3. Studies reporting partial clinical outcomes among children with HAdV mono- versus co-infection**

| **Author, year** | **Hospitalization** | | | **Pneumonia severity** | | **Deaths** |
| --- | --- | --- | --- | --- | --- | --- |
|  | **General hospitalization** | **Length of hospital stay** | **ICU admission** | **Common pneumonia** | **Severe pneumonia** |  |
| Aiping Chen, 2021 | No | No | No | No | Yes | No |
| Anna Franz, 2010 | No | No | No | Yes | Yes | No |
| Caiyun Wang, 2021 | No | Yes | No | No | Yes | No |
| Caiyun Wang, 2021 | No | Yes | No | No | Yes | No |
| Chunyan Liu, 2015 | No | No | Yes | No | Yes | No |
| Chunyang Jia, 2023 | No | No | No | No | Yes | No |
| Chunzhi Chen, 2020 | No | No | No | No | Yes | No |
| Dandan Jin, 2019 | No | No | No | No | Yes | No |
| Daoxiang Rong, 2020 | No | No | No | No | Yes | No |
| Débora Natalia Marcone, 2013 | Yes | No | No | No | No | No |
| Dinh-Dung Nguyen, 2023 | No | No | No | No | Yes | No |
| Enhui Xu, 2020 | No | No | No | No | Yes | No |
| Fuliang Jiang, 2022 | No | Yes | No | Yes | Yes | No |
| Ge Dai, 2020 | No | No | No | Yes | No | No |
| Hongming Che, 2021 | No | No | No | No | Yes | No |
| Hongwei Chen, 2022 | No | Yes | No | Yes | Yes | No |
| Hyun Jun Lee, 2016 | No | Yes | Yes | Yes | Yes | Yes |
| Jiahao Yuan, 2023 | No | No | No | Yes | No | No |
| Jiaotian Huang, 2021 | No | No | No | No | Yes | Yes |
| Jie Lin, 2020 | No | Yes | No | No | Yes | Yes |
| Jing Zhou, 2023 | No | No | No | No | Yes | No |
| Leyun Xie, 2018 | No | Yes | Yes | Yes | Yes | No |
| Leyun Xie, 2019 | No | No | Yes | No | Yes | No |
| Lifang Zhou, 2022 | No | Yes | No | Yes | Yes | Yes |
| Lingjian Zeng, 2021 | No | No | No | No | Yes | Yes |
| Lu Xu, 2021 | No | No | No | No | Yes | No |
| Maohua Li, 2022 | No | Yes | No | No | No | No |
| Marietjie Venter, 2011 | Yes | No | Yes | No | No | No |
| Mayda Finianos, 2016 | No | No | No | Yes | No | No |
| Miri Dotan, 2022 | No | No | Yes | No | No | No |
| Mo Chen, 2020 | No | No | No | No | Yes | No |
| P.Q. Huang, 2022 | No | Yes | Yes | No | No | Yes |
| Patricia Murtagh, 2009 | No | No | No | No | No | Yes |
| Peipei Du, 2020 | No | No | No | No | Yes | No |
| Qian Hu, 2021 | No | No | No | No | Yes | No |
| Qing Lou, 2021 | No | No | No | No | Yes | No |
| Quanheng Li, 2020 | No | No | No | Yes | Yes | No |
| Qun Lao, 2022 | No | Yes | No | No | No | No |
| Ran Liu, 2020 | No | No | No | No | No | Yes |
| Ruimu Zhang, 2021 | No | No | No | No | Yes | No |
| Sha Cai, 2023 | No | No | No | No | Yes | No |
| Sha Cai, 2023 | No | No | No | No | Yes | No |
| Shuangshuang Huang, 2023 | Yes | No | No | No | Yes | No |
| Silan Liu, 2021 | No | No | No | No | Yes | No |
| Sky Vanderburg, 2020 | No | No | Yes | No | Yes | Yes |
| Wanjun Li, 2018 | No | Yes | No | Yes | Yes | No |
| Wei Hou, 2022 | No | No | No | Yes | Yes | No |
| Xian Liu, 2021 | No | Yes | No | No | No | No |
| Xiaohua Zhou, 2017 | No | No | No | Yes | Yes | No |
| Xiaojun Kang, 2020 | No | No | No | No | Yes | No |
| Xiaoling Li , 2021 | No | No | No | Yes | No | No |
| Xiaoyan Sun, 2022 | No | No | No | No | Yes | No |
| Xinfen Yu, 2018 | Yes | No | No | No | No | No |
| Xiuping Liu, 2021 | No | No | No | No | Yes | No |
| Xuan Zheng, 2022 | No | Yes | No | Yes | Yes | No |
| Xueping Xu, 2020 | No | No | No | Yes | No | No |
| Yan Hong, 2021 | No | No | No | No | Yes | No |
| Yan Song, 2011 | No | No | No | No | Yes | No |
| Yang He, 2015 | Yes | No | No | No | No | No |
| Yanling Chen, 2021 | No | No | No | No | Yes | No |
| Yi Chen, 2014 | No | No | No | No | Yes | No |
| Yiman Huang, 2021 | No | Yes | No | No | No | No |
| Ying Li, 2022 | No | No | Yes | Yes | Yes | No |
| Yinyan Zhou, 2016 | No | No | No | Yes | No | Yes |
| Yuanmei Huang, 2017 | No | No | Yes | Yes | Yes | No |

Abbreviations: ICU=intensive care unit.

**Supplementary Table 4.** Summary of quality assessment for the studies reporting clinical outcomes among children with HAdV mono- versus co-infection

| **Author,**  **year of publication** | **Representativeness of the exposed cohort** | **Selection of the non exposed cohort** | **Ascertainment of exposure** | **Demonstration that outcome of interest was not present at start of study** | **Comparability of cohorts on the basis of the design or analysis** | **Assessment of outcome** | **Was follow-up long enough for outcomes to occur** | **Adequacy of follow up of cohorts** | **Score** | **Quality assessment** |
| --- | --- | --- | --- | --- | --- | --- | --- | --- | --- | --- |
| Aiping Chen, 2021 | 1 | 1 | 1 | 1 | 0 | 1 | 1 | 1 | 7 | High |
| Anna Franz, 2010 | 1 | 1 | 1 | 1 | 0 | 1 | 1 | 1 | 7 | High |
| Caiyun Wang, 2021 | 1 | 1 | 1 | 1 | 0 | 1 | 1 | 1 | 7 | High |
| Caiyun Wang, 2021 | 1 | 1 | 1 | 1 | 0 | 1 | 1 | 1 | 7 | High |
| Chunyan Liu, 2015 | 1 | 1 | 1 | 1 | 0 | 1 | 1 | 1 | 7 | High |
| Chunyang Jia, 2023 | 1 | 1 | 1 | 1 | 0 | 1 | 1 | 1 | 7 | High |
| Chunzhi Chen, 2020 | 1 | 1 | 1 | 1 | 1 | 1 | 0 | 1 | 7 | High |
| Dandan Jin, 2019 | 1 | 1 | 1 | 1 | 2 | 1 | 1 | 1 | 9 | High |
| Daoxiang Rong, 2020 | 1 | 1 | 1 | 1 | 0 | 1 | 1 | 1 | 7 | High |
| Débora Natalia Marcone, 2013 | 1 | 1 | 1 | 1 | 2 | 1 | 1 | 1 | 9 | High |
| Dinh-Dung Nguyen, 2023 | 1 | 1 | 1 | 1 | 0 | 1 | 0 | 1 | 6 | High |
| Enhui Xu, 2020 | 1 | 1 | 1 | 0 | 0 | 1 | 1 | 1 | 6 | High |
| Fuliang Jiang, 2022 | 1 | 1 | 1 | 1 | 0 | 1 | 0 | 1 | 6 | High |
| Ge Dai, 2020 | 1 | 1 | 1 | 0 | 0 | 1 | 1 | 1 | 6 | High |
| Hongming Che, 2021 | 1 | 1 | 1 | 1 | 0 | 1 | 1 | 1 | 7 | High |
| Hongwei Chen, 2022 | 1 | 1 | 1 | 1 | 0 | 1 | 1 | 1 | 7 | High |
| Hyun Jun Lee, 2016 | 1 | 1 | 1 | 1 | 1 | 1 | 1 | 1 | 8 | High |
| Jiahao Yuan, 2023 | 1 | 1 | 1 | 1 | 0 | 1 | 1 | 1 | 7 | High |
| Jiaotian Huang, 2021 | 1 | 1 | 1 | 1 | 0 | 1 | 1 | 1 | 7 | High |
| Jie Lin, 2020 | 1 | 1 | 1 | 1 | 0 | 1 | 1 | 1 | 7 | High |
| Jing Zhou, 2023 | 1 | 1 | 1 | 1 | 0 | 1 | 0 | 1 | 6 | High |
| Leyun Xie, 2018 | 1 | 1 | 1 | 1 | 1 | 1 | 1 | 1 | 8 | High |
| Leyun Xie, 2019 | 1 | 1 | 1 | 1 | 1 | 1 | 1 | 1 | 8 | High |
| Lifang Zhou, 2022 | 1 | 1 | 1 | 1 | 0 | 1 | 1 | 1 | 7 | High |
| Lingjian Zeng, 2021 | 1 | 1 | 1 | 1 | 0 | 1 | 1 | 1 | 7 | High |
| Lu Xu, 2021 | 1 | 1 | 1 | 0 | 1 | 1 | 1 | 1 | 7 | High |
| Maohua Li, 2022 | 1 | 1 | 1 | 1 | 0 | 1 | 1 | 1 | 7 | High |
| Marietjie Venter, 2011 | 1 | 1 | 1 | 1 | 1 | 1 | 1 | 1 | 8 | High |
| Mayda Finianos, 2016 | 1 | 1 | 1 | 1 | 0 | 1 | 1 | 1 | 7 | High |
| Miri Dotan, 2022 | 1 | 1 | 1 | 1 | 0 | 1 | 1 | 1 | 7 | High |
| Mo Chen, 2020 | 1 | 1 | 1 | 1 | 1 | 1 | 0 | 1 | 7 | High |
| P.Q. Huang, 2022 | 1 | 1 | 1 | 1 | 0 | 1 | 1 | 1 | 7 | High |
| Patricia Murtagh, 2009 | 1 | 1 | 1 | 1 | 1 | 0 | 1 | 1 | 7 | High |
| Peipei Du, 2020 | 1 | 1 | 1 | 1 | 0 | 1 | 1 | 1 | 7 | High |
| Qian Hu, 2021 | 1 | 1 | 1 | 1 | 1 | 1 | 1 | 1 | 8 | High |
| Qing Lou, 2021 | 1 | 1 | 1 | 1 | 1 | 1 | 0 | 1 | 7 | High |
| Quanheng Li, 2020 | 1 | 1 | 1 | 1 | 1 | 1 | 1 | 1 | 8 | High |
| Qun Lao, 2022 | 1 | 1 | 1 | 1 | 0 | 1 | 1 | 1 | 7 | High |
| Ran Liu, 2020 | 1 | 1 | 1 | 1 | 0 | 1 | 1 | 1 | 7 | High |
| Ruimu Zhang, 2021 | 1 | 1 | 1 | 1 | 0 | 1 | 0 | 1 | 6 | High |
| Sha Cai, 2023 | 1 | 1 | 1 | 1 | 1 | 1 | 1 | 1 | 8 | High |
| Sha Cai, 2023 | 1 | 1 | 1 | 1 | 0 | 1 | 1 | 1 | 7 | High |
| Shuangshuang Huang, 2023 | 1 | 1 | 1 | 1 | 0 | 1 | 1 | 1 | 7 | High |
| Silan Liu, 2021 | 1 | 1 | 1 | 0 | 0 | 1 | 1 | 1 | 6 | High |
| Sky Vanderburg, 2020 | 1 | 1 | 1 | 1 | 1 | 1 | 0 | 1 | 7 | High |
| Wanjun Li, 2018 | 1 | 1 | 1 | 1 | 0 | 1 | 1 | 1 | 7 | High |
| Wei Hou, 2022 | 1 | 1 | 1 | 1 | 0 | 1 | 1 | 1 | 7 | High |
| Xian Liu, 2021 | 1 | 1 | 1 | 1 | 0 | 1 | 1 | 1 | 7 | High |
| Xiaohua Zhou, 2017 | 1 | 1 | 1 | 1 | 0 | 1 | 1 | 1 | 7 | High |
| Xiaojun Kang, 2020 | 1 | 1 | 1 | 0 | 1 | 1 | 0 | 1 | 6 | High |
| Xiaoling Li, 2021 | 1 | 1 | 1 | 1 | 2 | 1 | 1 | 1 | 9 | High |
| Xiaoyan Sun, 2022 | 1 | 1 | 1 | 1 | 1 | 1 | 1 | 1 | 8 | High |
| Xinfen Yu, 2018 | 1 | 1 | 1 | 1 | 1 | 1 | 1 | 1 | 8 | High |
| Xiuping Liu , 2021 | 1 | 1 | 1 | 0 | 0 | 1 | 1 | 1 | 6 | High |
| Xuan Zheng, 2022 | 1 | 1 | 1 | 1 | 0 | 1 | 1 | 1 | 7 | High |
| Xueping Xu, 2020 | 1 | 1 | 1 | 1 | 2 | 1 | 1 | 1 | 9 | High |
| Yan Hong, 2021 | 1 | 1 | 1 | 1 | 1 | 1 | 1 | 1 | 8 | High |
| Yan Song, 2011 | 1 | 1 | 1 | 1 | 2 | 1 | 1 | 1 | 9 | High |
| Yang He, 2015 | 1 | 1 | 1 | 1 | 0 | 1 | 1 | 1 | 7 | High |
| Yanling Chen, 2021 | 1 | 1 | 1 | 0 | 1 | 1 | 0 | 1 | 6 | High |
| Yi Chen, 2014 | 1 | 1 | 1 | 0 | 1 | 1 | 1 | 1 | 7 | High |
| Yiman Huang, 2021 | 1 | 1 | 1 | 1 | 0 | 1 | 1 | 1 | 7 | High |
| Ying Li, 2022 | 1 | 1 | 1 | 1 | 0 | 1 | 1 | 1 | 7 | High |
| Yinyan Zhou, 2016 | 1 | 1 | 1 | 1 | 0 | 1 | 1 | 1 | 7 | High |
| Yuanmei Huang, 2017 | 1 | 1 | 1 | 1 | 0 | 1 | 1 | 1 | 7 | High |

Notes: High quality: 6-9 points.

**Supplementary Table 5.** Summary of findings on hospital stay duration (in days) among children with HAdV mono- versus co-infection

| **Studies** | **Mono-infection group** | **Co-infection group** | **Pathogens co-infected** | **Notes** |
| --- | --- | --- | --- | --- |
| Hyun Jun Lee, 2016 (18) | 5.0 (3.0, 54.0) | 6.0 (3.0, 9.0) | Any virus | Median (IQR) |
| Leyun Xie, 2018 (23) | 8.5 (6.0, 10.0) | 8.0 (6.0, 10.0) | Any virus | Median (IQR) |
| Qun Lao, 2022 (39) | 7.0 (5.0, 11.0) | 7.0 (5.0, 13.0) | Any virus | Median (IQR) |
| Xian Liu, 2021 (49) | 10.0 (8.0,12.0) | 9.6±2.6 | Any virus | Median (IQR)/Mean±SD |
| Leyun Xie, 2018 (23) | 8.5 (6.0, 10.0) | 10.0 (8.0, 13.0) | Any bacterium | Median (IQR) |
| Lifang Zhou, 2022 (25) | 6.0 (4.0,7.0) | 11.0 (6.5, 16.0) | Any bacterium | Median (IQR) |
| Qun Lao, 2022 (39) | 7.0 (5.0, 11.0) | 8.5 (6.0, 14.0) | Any bacterium | Median (IQR) |
| Wanjun Li, 2018 (47) | 7.2±1.8 | 7.0±1.8 | Any bacterium | Mean±SD |
| Xian Liu, 2021 (49) | 10.0 (8.0,12.0) | 13.0 (8.0,17.5) | Any bacterium | Median (IQR) |
| Leyun Xie, 2018 (23) | 8.5 (6.0, 10.0) | 7.0 (6.0, 8.3) | MP | Median (IQR) |
| Wanjun Li, 2018 (47) | 7.2±1.8 | 7.2±1.7 | MP | Mean±SD |
| Ying Li, 2022 (64) | 9.0（7.0,13.0） | 11.0（8.0,13.0） | MP | Median (IQR) |
| Hyun Jun Lee, 2016 (18) | 5.0 (3.0, 54.0) | 6 (3, 7) | RSV | Median (IQR) |
| Wanjun Li, 2018 (47) | 7.2±1.8 | 6.6±1.4 | RSV | Mean±SD |
| Hyun Jun Lee, 2016 (18) | 5.0 (3.0, 54.0) | 5.0 (4.0, 7.0) | RhV | Median (IQR) |
| Wanjun Li, 2018 (47) | 7.2±1.8 | 7.7±3.0 | CMV | Mean±SD |
| Wanjun Li, 2018 (47) | 7.2±1.8 | 11.5±1.3 | EB | Mean±SD |
| Wanjun Li, 2018 (47) | 7.2±1.8 | 7.0±1.8 | RV | Mean±SD |
| Xian Liu, 2021 (49) | 10.0 (8.0, 12.0) | 9.0 (8.0, 14.0) | Any atypical pathogen | Median (IQR) |
| P.Q. Huang, 2022 (33) | 10.0 (8.0, 13.0) | 18.0 (13.0, 28.0) | Any fungus | Median (IQR) |
| Qun Lao, 2022 (39) | 7.0 (5.0, 11.0) | 9.0 (6.0, 15.0) | Chlamydia | Median (IQR) |

Abbreviations: HAdV=human adenovirus, MP=mycoplasma pneumoniae, RSV=respiratory syncytial virus, RhV=rhinovirus, CMV=cytomegalovirus, EB=epstein-barr virus, RV=rotavirus, IQR=interquartile range, SD=standard deviation.


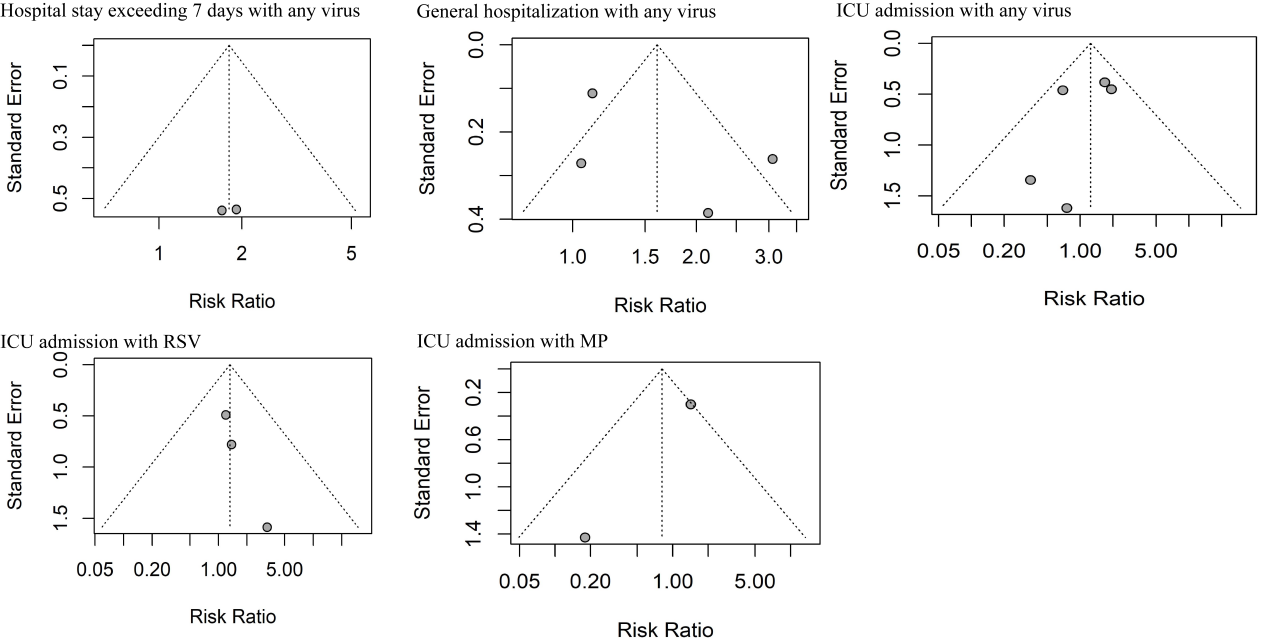


**Supplementary Figure 1.** Funnel plots illustrating the relationship between hospitalization and HAdV co-infection with any virus, MP, and RSV in children.

Abbreviations: ICU=intensive care unit, MP=mycoplasma pneumoniae, RSV=respiratory syncytial virus.


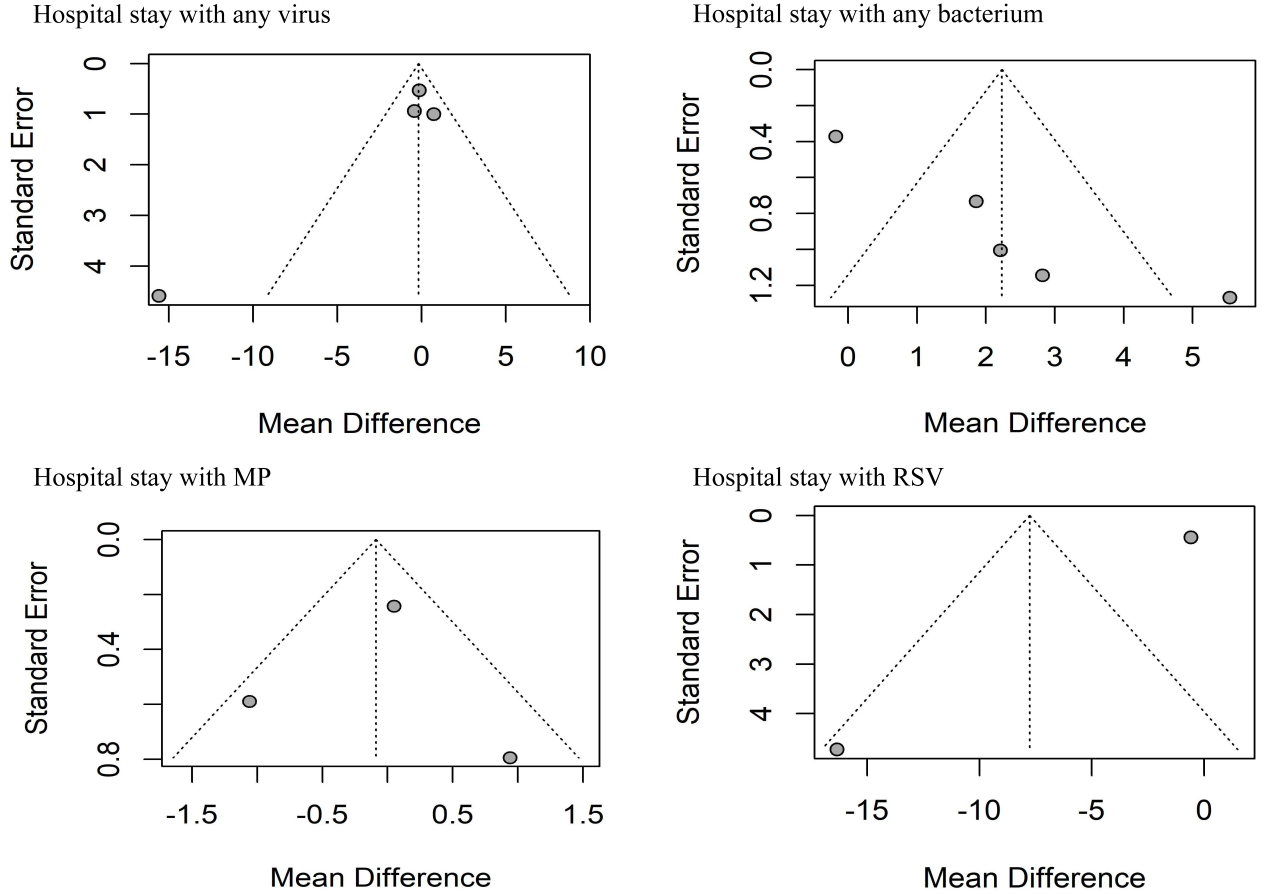


**Supplementary Figure 2.** Funnel plots illustrating the relationship between hospital stay and HAdV co-infection with any virus, bacterium, MP, and RSV in children.

Abbreviations: MP=mycoplasma pneumoniae, RSV=respiratory syncytial virus.

**
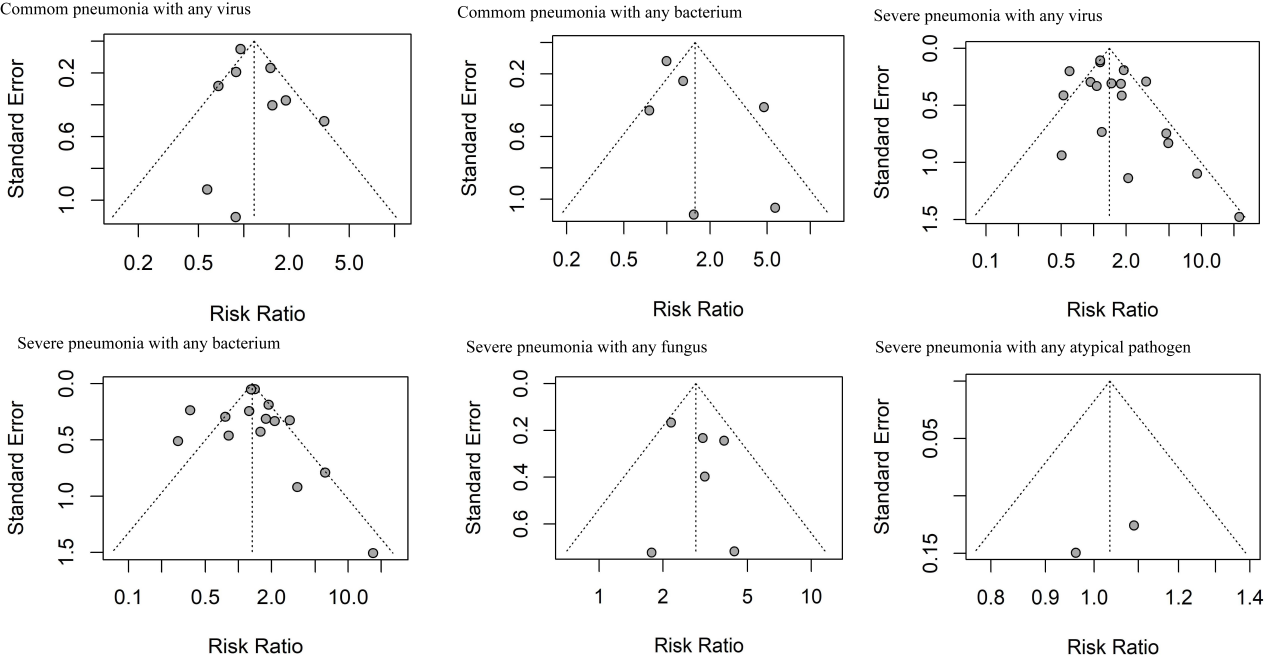
**

**Supplementary Figure 3.** Funnel plots illustrating the relationship between pneumonia severity and HAdV co-infection with any virus, bacterium, fungus, or atypical pathogen in children.


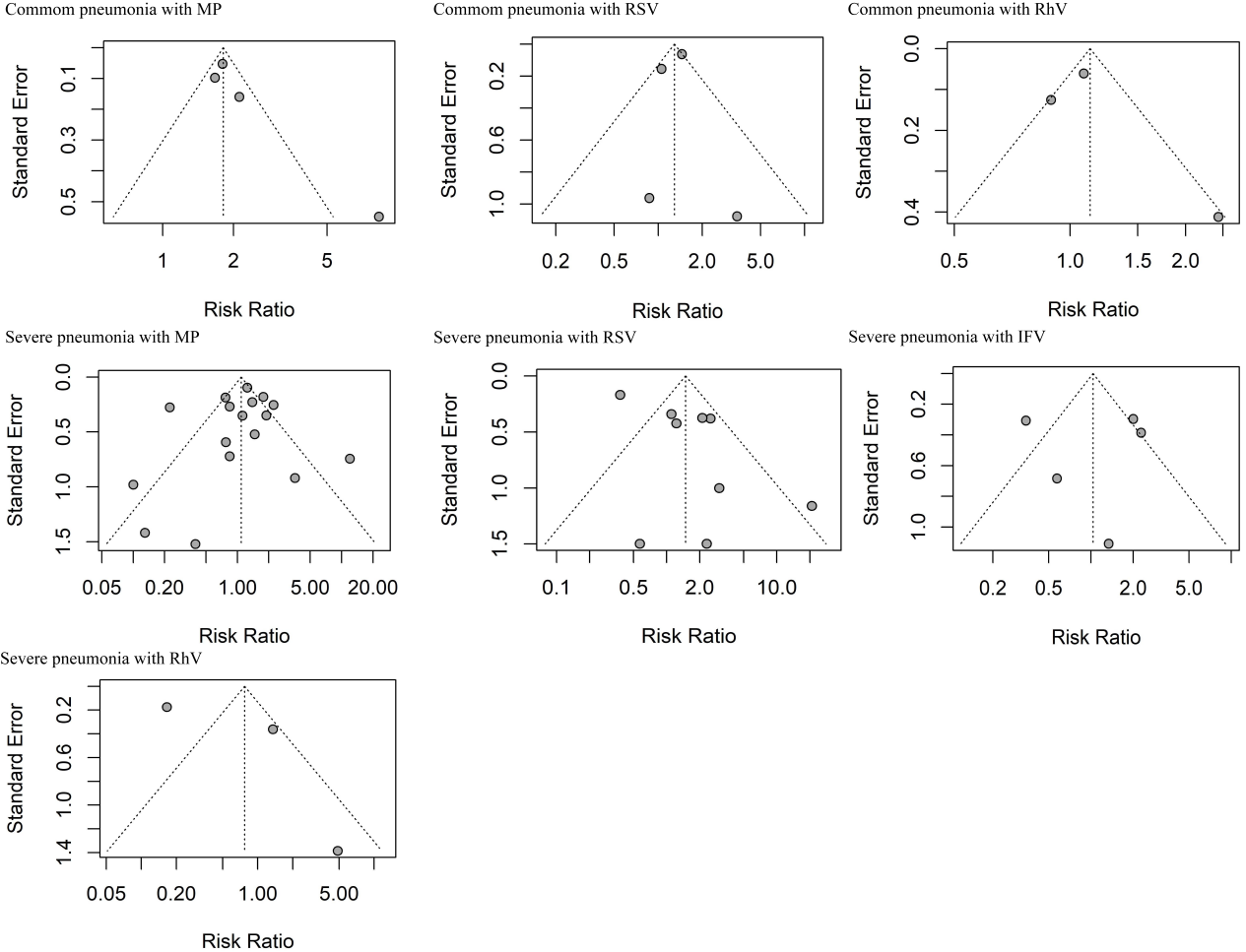


**Supplementary Figure 4.** Funnel plots illustrating the relationship between pneumonia severity and HAdV co-infection with single pathogen in children.

Abbreviations: MP=mycoplasma pneumoniae, RSV=respiratory syncytial virus, RhV=rhinovirus, IFV=influenza virus.


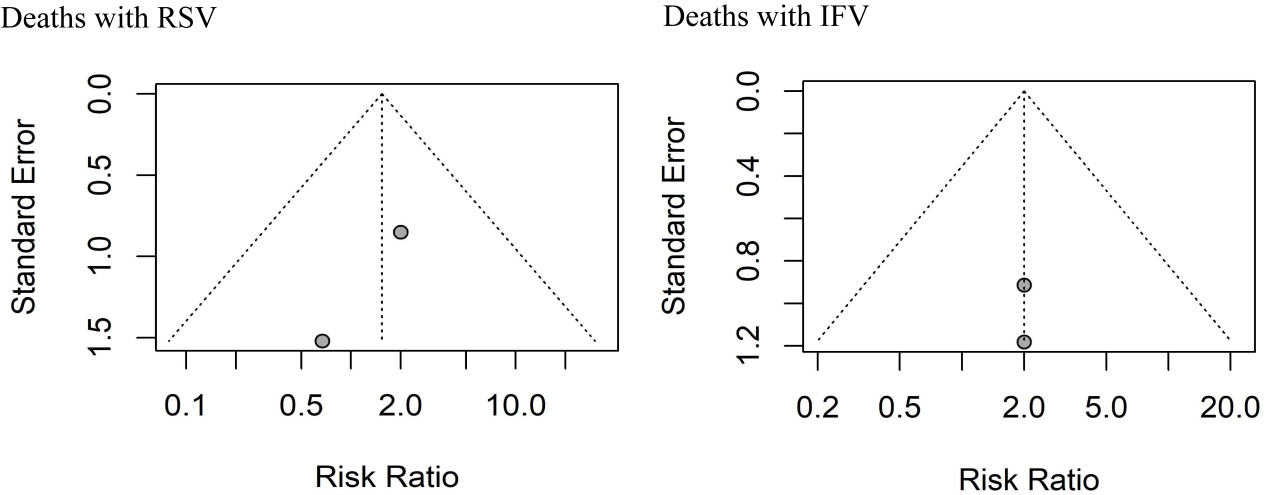


**Supplementary Figure 5.** Funnel plots illustrating the relationship between deaths and HAdV co-infection with RSV and IFV in children.

Abbreviations: RSV=respiratory syncytial virus, IFV=influenza virus.

**Supplementary Table 6.** Results of Begg's and Egger's test in a meta-analysis of the effects of adenovirus co-infection with other pathogens on clinical outcomes

| **Clinical outcomes** | | **Pathogens HAdV co-infection with** | **Number of studies** | **Begg's test** | | **Egger's test** | |
| --- | --- | --- | --- | --- | --- | --- | --- |
|  |  |  |  | **Z value** | **P value** | **T value** | **P value** |
| Hospitalization | General hospitalization | Any virus | 4 | 0.34 | 0.734 | 1.20 | 0.354 |
|  | Hospital stay | Any virus | 4 | -0.34 | 0.734 | -1.74 | 0.225 |
|  |  | Any bacterium | 5 | 1.71 | 0.086 | 5.93 | 0.010 |
|  |  | MP | 3 | 0.00 | 1.000 | -0.10 | 0.937 |
|  |  | RSV | 2 | － | － | － | － |
|  | Hospital stays exceeding 7 days | Any virus | 2 | － | － | － | － |
|  | ICU admission | Any virus | 5 | -0.24 | 0.807 | -0.81 | 0.479 |
|  |  | RSV | 3 | 1.04 | 0.296 | 3.53 | 0.176 |
|  |  | MP | 2 | － | － | － | － |
| Pneumonia severity | Common pneumonia | Any virus | 7 | 0.52 | 0.602 | 1.20 | 0.268 |
|  |  | Any bacterium | 6 | 0.38 | 0.707 | 1.39 | 0.236 |
|  |  | MP | 4 | 1.02 | 0.308 | 1.95 | 0.191 |
|  |  | RSV | 4 | 0.00 | 1.000 | -0.34 | 0.766 |
|  |  | RhV | 4 | 0.00 | 1.000 | 0.48 | 0.717 |
|  | Severe pneumonia | Any virus | 18 | 1.21 | 0.226 | 1.58 | 0.134 |
|  |  | Any bacterium | 15 | 0.59 | 0.553 | 0.02 | 0.980 |
|  |  | Any fungus | 6 | 0.00 | 1.000 | 0.64 | 0.556 |
|  |  | Any atypical pathogen | 2 | － | － | － | － |
|  |  | MP | 17 | -0.95 | 0.343 | -0.49 | 0.230 |
|  |  | RSV | 9 | 0.10 | 0.920 | 2.52 | 0.040 |
|  |  | IFV | 5 | -0.24 | 0.807 | 0.01 | 0.992 |
|  |  | RhV | 3 | 0.00 | 1.000 | 1.25 | 0.431 |
| Deaths | | RSV | 2 | － | － | － | － |
|  |  | IFV | 2 | － | － | － | － |

Abbreviations: HAdV=human adenovirus, MP=mycoplasma pneumoniae, RSV=respiratory syncytial virus, RhV=rhinovirus, IFV=influenza virus; “－” referred to the meta-analyses with less than 2 studies without Begg's and Egger's test; A P value greater than 0.5 was considered statistically significant.

**
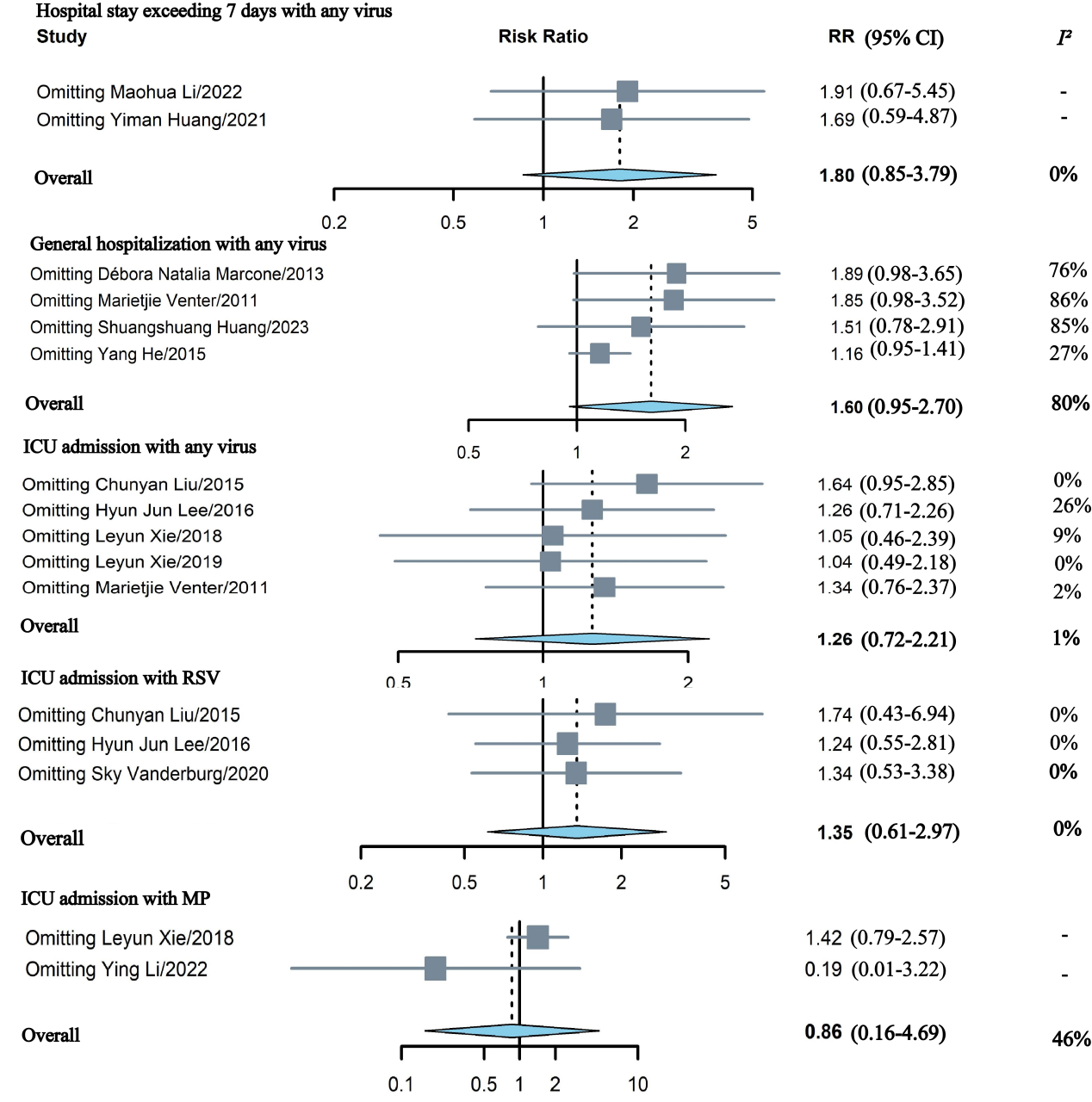
**

**Supplementary Figure 6.** Forest plots illustrating the relationship between hospitalization and HAdV co-infection with any virus, MP, and RSV in children.

Abbreviations: RR=risk ratios, RSV=respiratory syncytial virus, MP=mycoplasma pneumoniae, ICU=intensive care unit.

**
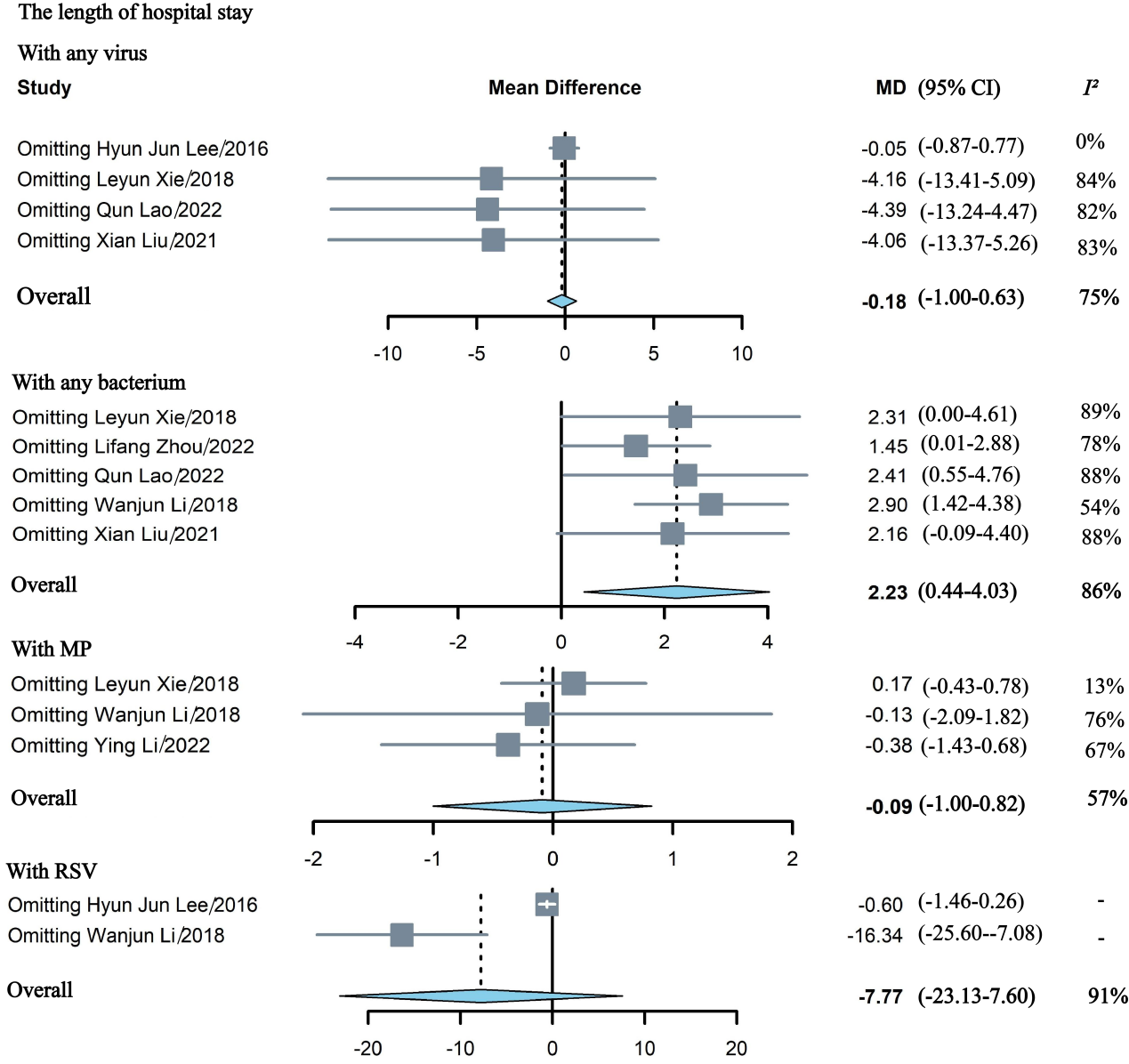
**

**Supplementary Figure 7.** Forest plots illustrating the relationship between the length of hospital stay and HAdV co-infection with any virus, bacterium, MP, and RSV in children.

Abbreviations: MD=mean difference, RSV=respiratory syncytial virus, MP=mycoplasma pneumoniae.

**
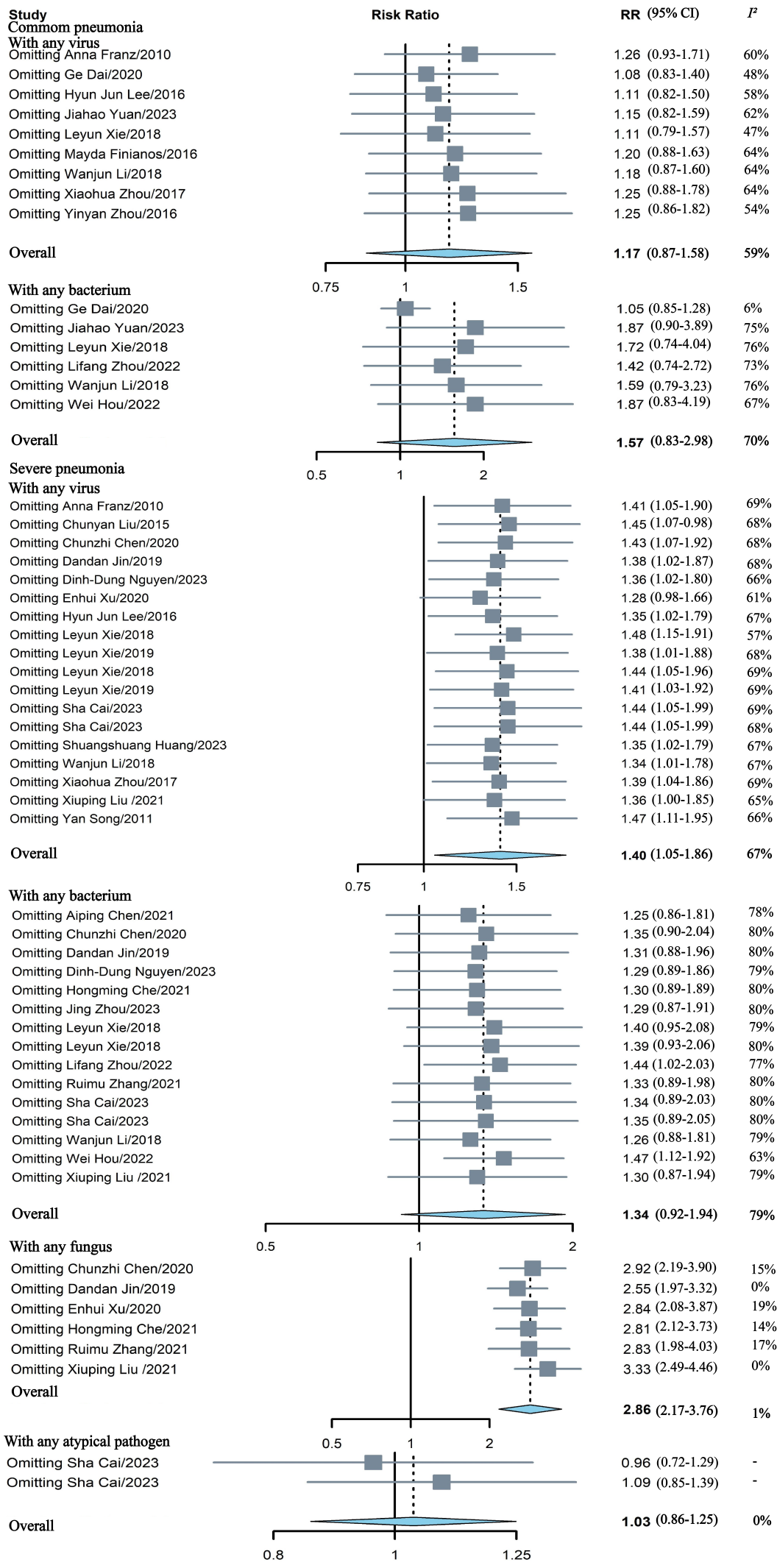
**

**Supplementary Figure 8.** Forest plots illustrating the relationship between pneumonia severity and HAdV co-infection with any virus, bacterium, fungus, or atypical pathogen in children.

Abbreviations: RR=risk ratios.

**
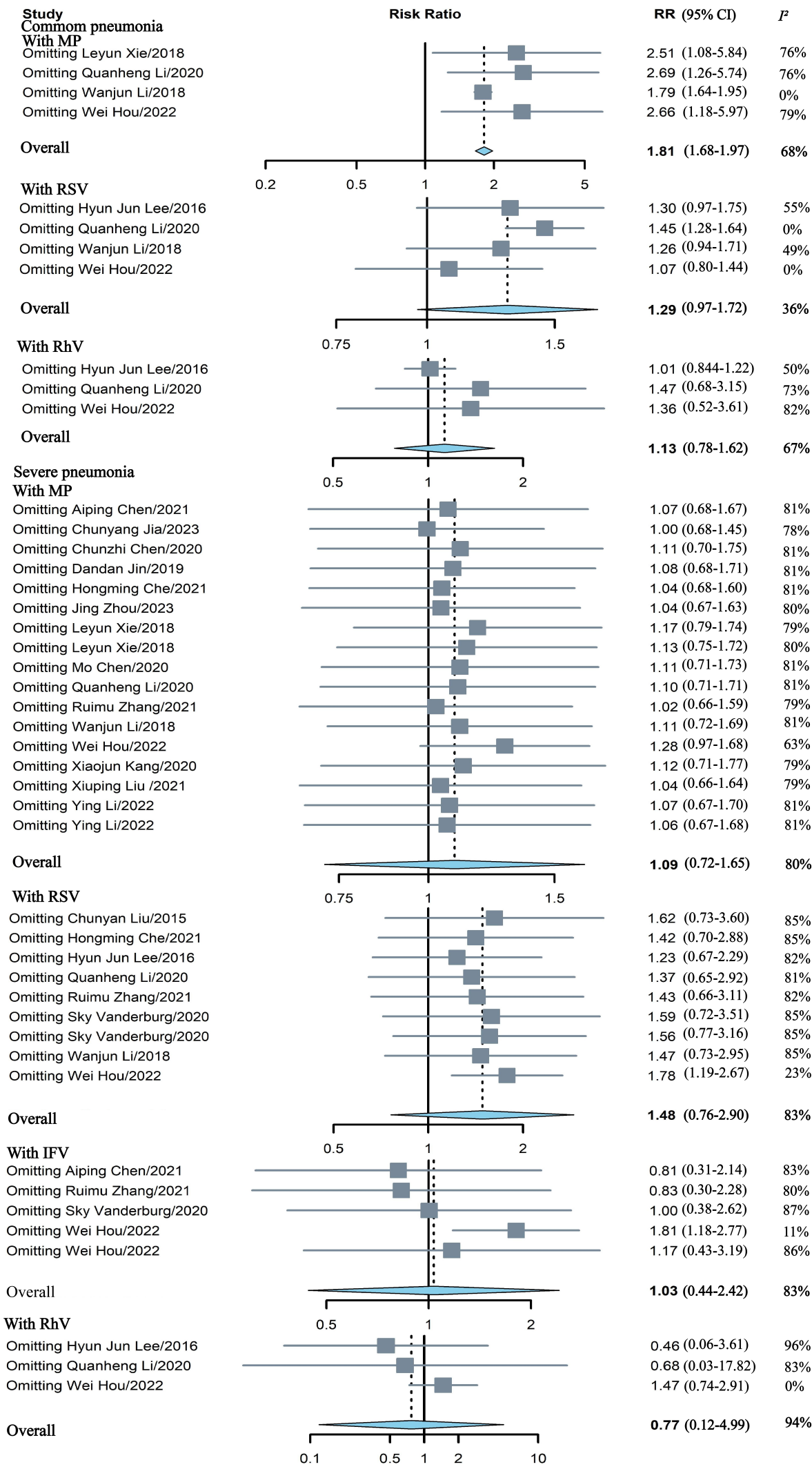
**

**Supplementary Figure 9.** Forest plots illustrating the relationship between pneumonia severity and HAdV co-infection with single pathogen in children.

Abbreviations: RR=risk ratios, MP=mycoplasma pneumoniae, RSV=respiratory syncytial virus, RhV=rhinovirus, IFV=influenza virus.

**
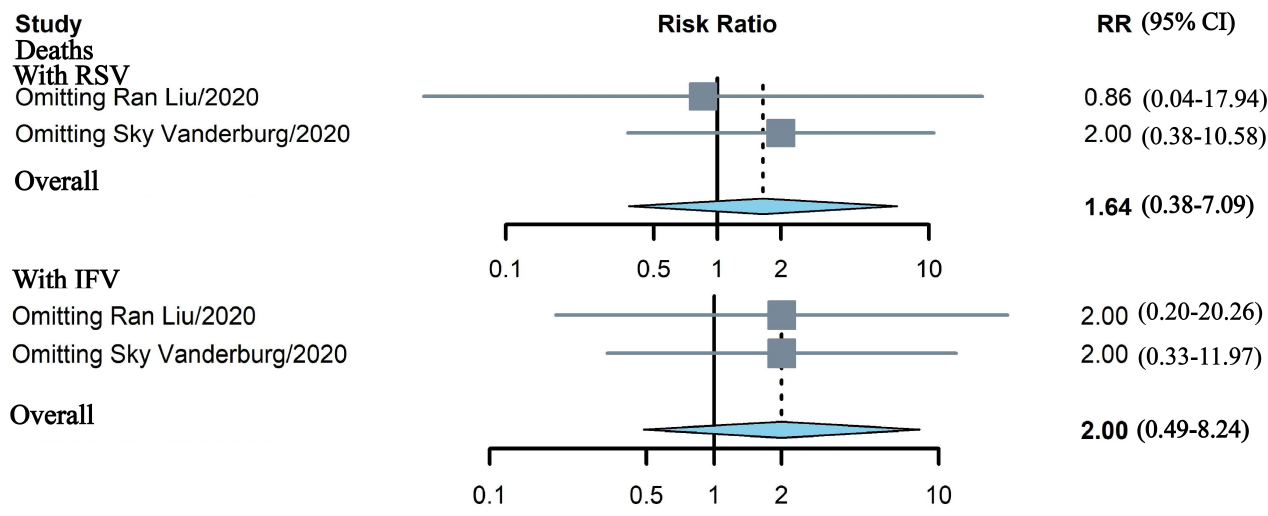
**

**Supplementary Figure 10.** Forest plots illustrating the relationship between deaths and HAdV co-infection with RSV and IFV in children.

Abbreviations: RR=risk ratios, RSV=respiratory syncytial virus, IFV=influenza virus.

**
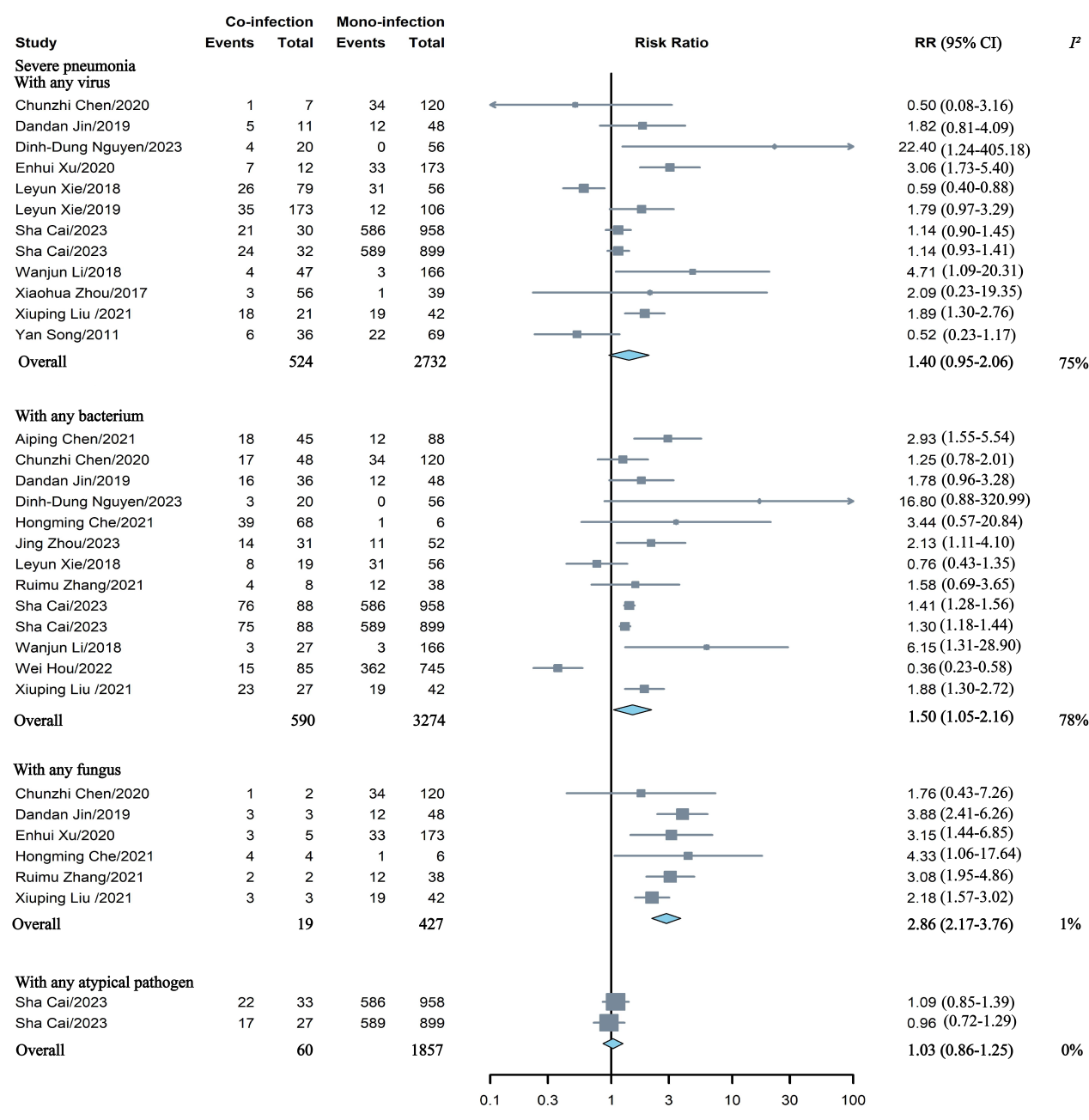
**

**Supplementary Figure 11.** Forest plots illustrating the relationship between severe pneumonia and HAdV co-infection with any virus, bacterium, fungus, and atypical pathogen in children.

Abbreviations: RR=risk ratios.

**REFERENCES**

1. Vrabel M. Preferred reporting items for systematic reviews and meta-analyses: the PRISMA statement. *Revista Espaola De Nutrición Humana Y Dietética*. 2009;18(3):e123.

2. Chen A, Xiao Z, Xiao Z, et al. Investigation of the clinical characteristies of adenovirus pneumoniain children and Logistie regression analysis of risk factors. *J Community Med*. 2021;19(18):1096-1100.

3. Franz A, Adams O, Willems R, et al. Correlation of viral load of respiratory pathogens and co-infections with disease severity in children hospitalized for lower respiratory tract infection. *J Clin Virol*. 2010;48(4):239-245.

4. Wang C, Liu J, Mi Y, et al. Clinical features and epidemiological analysis of respiratory human adenovirus infection in hospitalized children: a cross-sectional study in Zhejiang. *Virology Journal*. 2021;18(1):234.

5. Wang C, Liu J, Mi Y, et al. Clinical and epidemiological characteristics of respiratory adenovirus infections in children: analysis of 488 cases. *Chin J Clin Infect Dis*. 2021;14(2):121-126.

6. Liu C, Xiao Y, Zhang J, et al. Adenovirus infection in children with acute lower respiratory tract infections in Beijing, China, 2007 to 2012. *BMC Infect Dis*. 2015;15(1):408.

7. Jia C, Shi S. CT findings and clinical features of adenovirus pneumonia in children. *J Clin Radiol*. 2023;42(3):472-476.

8. Chen C, Lou Q. Clinical characteristics and severe risk factors of mixed infection with adenovirus pneumonia in children. *Chin J of Clinical Rational Drug Use*. 2020;13(11):133-135.

9. Jin D. Characteristics of mixed infection of adenovirus pneumonia in children and risk factors of severe cases. *Journal of Clinical Pulmonary Medicine*. 2019;24(10):1747-1750.

10. Rong D, Xuan A, Fang S, et al. Clinical characteristics of 120 children with adenovirus pneumonia. *Chinese Journal of General Practice*. 2020;18(7):1096-1142.

11. Marcone DN, Ellis A, Videla C, et al. Viral etiology of acute respiratory infections in hospitalized and outpatient children in Buenos Aires, Argentina. *Pediatr Infect Dis J*. 2013;32(3):e105-110.

12. Nguyen DD, Phung LT, Thanh Tran HT, et al. Molecular subtypes of respiratory Adenovirus infection outbreak in children in Northern Vietnam and risk factors of more severe cases. *PLoS Negl Trop Dis*. 2023;17(11):e0011311.

13. Xu E, Yuan L, Zhuo Z. Risk factors for severe adenovirus pneumonia in children. *Chinese Journal of Practical Medicine*. 2020;47(24):19-21.

14. Jiang F, Pan W, Lin Y. Clinical characteristics of mixed infection in children with acute respiratory adenovirus infection. *Chinese and Foreign Medical Research*. 2022;20(07):78-82.

15. Dai G, Wang T, Jiang W, et al. Clinical analysis of 37 cases of bronchiolitis obliterans after adenovirus pneumonia. *Chin J Appl Clin Pediatr*. 2020;35(16):1235-1238.

16. Che H. Analysis of risk factors and evaluation of serious complications under different drugs therapy of severe adenovirus pneumonia in children: Jilin University; 2021.

17. Chen H. Analysis of clinical characteristics of adenovirus respiratory infection and risk factors of severe adenoviral pneumonia in children: Huazhong University of Science and Technology; 2022.

18. Lee HJ, Seo YE, Han SB, et al. Clinical impact of mixed respiratory viral infection in children with adenoviral infection. *Infect Chemother*. 2016;48(4):309-316.

19. Yuan J, Wei M, Chen M, et al. Risk factors for the development of bronchiolitis obliterans in children after suffering from adenovirus pneumonia. *Frontiers in Pediatrics*. 2023;11(1):1335543.

20. Huang J, Lu X, Zhu Y, et al. Risk factors for mixed infections and clinical characteristics in children with severe adenovirus pneumonia. *Chin Pediatr Emerg Med*. 2021;28(9):756-762.

21. Lin J, Cao Q, Li B, et al. Clinical characteristics and prevalence of adenovirus with respiratory tract infections in children. *Chin Pediatr Emerg Med*. 2020;27(2):114-118.

22. Zhou J, Lv Y, Han Y, et al. Analysis of influencing factors of severe adenovirus pneumonia in children. *China modern medicine*. 2023;30(3):109-112.

23. Xie L, Zhang B, Zhou J, et al. Human adenovirus load in respiratory tract secretions are predictors for disease severity in children with human adenovirus pneumonia. *Virol J*. 2018;15(1):123.

24. Xie L, Zhang B, Xiao N, et al. Epidemiology of human adenovirus infection in children hospitalized with lower respiratory tract infections in Hunan, China. *J Med Virol*. 2019;91(3):392-400.

25. Zhou L, Ouyang Z, Hao C, et al. Clinical features of severe adenovirus pneumonia with bacterial infection in children. *Chin Pediatr Emerg Med*. 2022;29(6):446-450.

26. Zeng L, Wei J, Tang Y, et al. Clinical characteristics of human adenovirus plastic bronchitis in 10 pediatric cases: a retrospective study of seven years. *Virologica Sinica*. 2021;36(3):550-554.

27. Xu L, Cao J, Pan J. Risk factors of severe adenovirus pneumonia in children. *Journal of Clinical Pulmonary Medicine*. 2021;26(8):1169-1175.

28. Li M, Liu Q, Zhang Y, et al. Human adenovirus pulmonary infection in 84 children and DNA genotypes. *Chin J Nosocomiol*. 2022;32(24):3764-3766.

29. Venter M, Lassaunière R, Kresfelder TL, et al. Contribution of common and recently described respiratory viruses to annual hospitalizations in children in South Africa. *J Med Virol*. 2011;83(8):1458-1468.

30. Finianos M, Issa R, Curran MD, et al. Etiology, seasonality, and clinical characterization of viral respiratory infections among hospitalized children in Beirut, Lebanon. *J Med Virol*. 2016;88(11):1874-1881.

31. Dotan M, Zion E, Bilavsky E, et al. Adenovirus can be a serious, life-threatening disease, even in previously healthy children. *Acta Paediatrica*. 2022;111(3):614-619.

32. Chen M, Kou L, Liu H. Characteristics of adenovirus pneumonia in children and risk factors of severe pneumonia. *Chinese and Foreign Medical Research*. 2020;18(25):55-57.

33. Huang PQ, Du H, Chen HB, et al. Invasive pulmonary fungal infections in children with severe human adenovirus type 7 pneumonia: A retrospective study. *Pediatr Neonatol*. 2022;63(4):388-393.

34. Murtagh P, Giubergia V, Viale D, et al. Lower respiratory infections by adenovirus in children. Clinical features and risk factors for bronchiolitis obliterans and mortality. *Pediatr Pulmonol*. 2009;44(5):450-456.

35. Du P. Epidemiological of respiratory adenovirus and clinical characteristics of adenovirus pneumonia in hospitalized children in Suzhou area: SooChow University; 2020.

36. Hu Q, Zheng Y, Wang W, et al. Clinical feature analysis of 541 children with adenovirus pneumonia. *Chin J Appl Clin Pediatr*. 2021;36(16):1230-1234.

37. Lou Q, Zhang S-x, Yuan L. Clinical analysis of adenovirus pneumonia with pulmonary consolidation and atelectasis in children. *Journal Of International Medical Research*. 2021;49(2):0300060521990244.

38. Li Q, Hao X, Dong W, et al. Epidemiological and clinical characteristies analysis of adenovirus infection in 9962 hospitalized children with acute respiratory tract infection. *CJChc Dec*. 2020;28(12):1412-1415.

39. Lao Q, Han N, Pan H, et al. Identified risk factors for co-infection in hospitalised children infected with adenovirus in Hangzhou. *Epidemiol Infect*. 2022;150(1):1-19.

40. Liu R. Analysis of risk factors for death of severe pneumonia with acute respiratory failure in PICU: Nanchang University; 2020.

41. Zhang R, Wang H, Tian S, et al. Adenovirus viremia may predict adenovirus pneumonia severity in immunocompetent children. *BMC Infect Dis*. 2021;21(1):213.

42. Cai S, Zhu C, Chen R, et al. Risk factors of severe adenoviruss pneumonia in children. *Chin J Clin Res*. 2023;36(4):568-571.

43. Cai S, Chen R, Gao M, et al. Construction and validation of risk score model of severe adenovirus pneumonia in children. *China modern medicine*. 2023;30(18):93-105.

44. Huang S, Wang H, Li L, et al. Molecular epidemiology and phylogenetic analyses of human adenovirus in pediatric patients with acute respiratory infections from Hangzhou during COVID-19 pandemic. *Front Pediatr*. 2023;11(1):1237074.

45. Liu S. Study on the correlation between adenovirus pneumonia and IFN-λ1 mRNA expression: Hunan Normal University; 2021.

46. Vanderburg S, Wijayaratne G, Danthanarayana N, et al. Outbreak of severe acute respiratory infection in Southern Province, Sri Lanka in 2018: A cross-sectional study. *BMJ Open*. 2020;10(11):e040612.

47. Li W. The investigation of epidemiological chasracteristics, clinical features and mixed infection of adenovirus infection in Tai 'an area: Shandong First Medical University; 2018.

48. Hou W, Zhang L, Zhang M, et al. Epidemiological investigation of adenovirus respiratory tract infection in hospitalized children. *Clinical Focus*. 2022;37(10):916-920.

49. Liu X. Clinical characteristics with severe adenovirus pneumonia and relationship between serum c-reactive protein to serum albumin radio and the prognosis in children: Jishou University; 2021.

50. Zhou X, Zhao Y, Lin G, et al. Epidemiological and clinical analysis of children with respiratory tract infection of adenovirus in chaoshan area. *Int J Respir*. 2017;37(10):745-751.

51. Kang X, Sui X, Zhuo Z. Predictors of pneumonia in children severe adenovirus. *Chin Pediatr Integr Tradit West Med*. 2020;12(6):534-537.

52. Li X, He W, Shi P, et al. Risk factors of bronchiolitis obliterans after adenovirus pneumonia: A nested case-control study. *Chin J Evid Based Pediatr*. 2021;16(3):233-236.

53. Sun X, Zhao S, Wang X. Construction and validation of a nomogram for predicting the severity of adenovirus pneumonia in children. *Chin J Exp Clin Infect Dis (Electronic Edition)*. 2022;16(5):337-343.

54. Yu X, Kou Y, Zhou Y, et al. Analysis of pathogen characteristics in children with acute respiratory tract infection. *Chinese J Exp Clin Virol*. 2018;32(2):160-165.

55. Liu X, Wang Y. Analysis of risk factors and diagnostic value of LDH in children with severe adenovirus pneumonia. *Medical Innovation of China*. 2021;18(6):68-72.

56. Zheng X, Guo P, Lei Z, et al. Clinical and epidemiological characteristics of human adenovirus in hospitalized children with respiratory tract infections in Henan province. *International Journal of Virology*. 2022;29(6):516-520.

57. Xu X, Tian M, Gu W, et al. Clinical characteristics of severe adenovirus pneumonia and risk factors of concurrent bronchiolitis obliterans. *Chinese Journal of Practical Pediatrics*. 2020;35(12):968-972.

58. Hong Y, Song Y, Wang Q, et al. Risk factors analysis of severe adenovirus pneumonia in children. *Journal of Gannan Medical University*. 2021;41(7):688-711.

59. Song Y. Prevalence and clinical features of adenovirus infection with respiratory tract infection in hospitalized children in Chongqing: Chongqing Medical University; 2011.

60. He Y. Analysis of respiratory virus infection characteristics in outpatient and inpatient children in a certain hospital from 2011 to 2012. *Maternal and child health care of China*. 2015;30(3):382-384.

61. Chen Y, Chen L, Chen Y, et al. Clinical characteristics and early recognition of severe adenovirus pneumonia in children. *Fujian Med J*. 2021;43(02):34-37.

62. Chen Y, Lian G, Zhang Y, et al. Etiology of community-acquired pneumonia among pediatric inpatients in Guangzhou during 2012 and 2013. *Chin J Clin Infect Dis*. 2014;7(6):521-525.

63. Huang Y, Wang C, Ma F, et al. Human adenoviruses in paediatric patients with respiratory tract infections in Beijing, China. *Virol J*. 2021;18(1):191.

64. Li Y, Chen H, Hu R, et al. Clinical analysis of 36 children with pneumonia caused by coinfection of human adenovirus type 7 and Mycoplasma pneumoniae. *Chin J Appl Clin Pediatr*. 2022;37(8):611-614.

65. Zhou Y, Yu X, Kou Y, et al. Detection and genetie evolution of adenovirus from children with acute respiratory tract infections. *Chin J Clin Infect Dis*. 2016;9(1):24-31.

66. Huang Y. Clinical study on adenovirus respiratory tract infection in children in Suzhou: SooChow University; 2017.
